# Comparative transcriptomic and metabolomic analysis reveals pectoralis highland adaptation across altitudinal songbirds

**DOI:** 10.1111/1749-4877.12620

**Published:** 2022-01-10

**Authors:** Ying XIONG, Yan HAO, Yalin CHENG, Liqing FAN, Gang SONG, Dongming LI, Yanhua QU, Fumin LEI

**Affiliations:** ^1^ Key Laboratory of Zoological Systematics and Evolution Institute of Zoology Chinese Academy of Sciences Beijing China; ^2^ University of Chinese Academy of Sciences Beijing China; ^3^ Key Laboratory of Forest Ecology in Tibet Plateau of Ministry of Education Institute of Tibet Plateau Ecology Tibet Agriculture & Animal Husbandry University Nyingchi China; ^4^ Key Laboratory of Animal Physiology, Biochemistry and Molecular Biology of Hebei Province, College of Life Sciences Hebei Normal University Shijiazhuang China; ^5^ Center for Excellence in Animal Evolution and Genetics Chinese Academy of Sciences Kunming China

**Keywords:** altitude, gas chromatography–mass spectrometry, hypoxia, pectoralis, RNA–seq

## Abstract

Pectoralis phenotypic variation plays a fundamental role in locomotion and thermogenesis in highland birds. However, its regulatory and metabolic mechanisms remain enigmatic to date. Here, we integrated phenomic, transcriptomic, and metabolomic approaches to determine muscle variation and its underpinning mechanisms across altitudinal songbirds. Phenomics confirmed that all highland birds had considerable increases in muscle oxidative capacity, capillarity, and mitochondrial abundance in our study. Correspondingly, transcriptomic analyses found that differentially expressed genes in phenotype‐associated modules enriched for blood vessel, muscle structure development, and mitochondrial organization. Despite similar traits and functional enrichments across highland birds, different mechanisms drove their occurrence in high‐altitude tree sparrow and 2 snow finches. Importantly, a metabolic feature shared by all the 3 highland birds is the improvement in insulin sensitivity and glucose utilization through activating insulin signaling pathway, which is vital to increase muscle oxidative capacity and maintain metabolic homeostasis. Nevertheless, fatty acid biosynthesis and oxidation are enhanced in only 2 snow finches which had a long evolutionary history on the high plateau, also differing from ketone body metabolism in recently introduced colonizer of the tree sparrow of the high plateau. Our study represents a vital contribution to reveal the regulatory and metabolic basis of pectoralis variation across altitudinal songbirds.

## Graphical Abstract

Co‐expression analyses of differentially expressed genes and muscle traits via WGCNA reveal potential genetic basis of muscle development, angiogenesis, and mitochondrial organization.

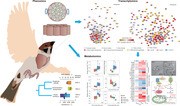

## INTRODUCTION

At high altitude, hypoxia and hypothermia are 2 significantly severe challenges to aerobic exercise and thermogenesis in small endotherms, because a high rate of O_2_ flux should be concurrently sustained to thermogenesis in cold temperature (Scott *et al*. 2015). Therefore, physiological modifications, including typically greater capillary density, more oxidative fiber, and higher proportion of subsarcolemmal mitochondria have been found in pectoralis of highland birds (Scott *et al*. 2009; Xiong *et al*. 2020). Understanding the molecular and biochemical mechanism of pectoralis modification is essential to investigate the functional evolution of phenotypic variation under hypoxic hypothermia. However, the regulatory and metabolic basis of muscle phenotype remains largely unknown for highland birds, in particular resident species with different evolutionary history.

In birds, pectoralis muscle is a fundamental tissue for locomotion and thermogenesis, while different fiber types (e.g. slow oxidative (SO), fast oxidative glycolytic (FOG), and fast glycolytic (FG) fiber) have been suggested to perform diverse functions due to their various contractile speed and metabolic capacity (Schiaffino & Reggiani 2011). Therefore, adaptive modifications in pectoralis of highland migrant birds are mainly reflected in changes in the relative proportion of fiber types (Scott *et al*. 2009). Previous studies, however, demonstrated that the pectoralis muscle of most small songbirds comprised exclusively FOG fibers (Marquez *et al*. 2006; Xiong *et al*. 2020). Thereby, small songbirds would take an alternative way to transform muscle phenotype but not to change muscle fiber types for responding to highland environmental stress. Moreover, resident small songbirds with different evolutionary time in the highland might be proposed to evolve various muscle characteristics.

Previous work mainly focused on uncovering bird genomic divergence to high altitude adaptation, while the role of the changes in gene expression contributing to muscle variation is given less attention (Qu *et al*. 2013, 2020). In high‐altitude rodents, the oxidative phenotype and capillarity of the gastrocnemius are related to the differential expression of genes involved in energy metabolism, muscle plasticity, and cell stress response (Scott *et al*. 2015). Hence, transcriptomic responses in pectoralis can prove an important insight into genes and biochemical pathways involved in trait variation or evolutionary adaptation to highland environment (Scott *et al*. 2015). Gene expression networks and key genes associated with phenotype are identified through weighted gene coexpression network analysis (WGCNA) in previous studies (Velotta *et al*. 2018; Hao *et al*. 2019). Likewise, skeletal muscle plays a central role on whole‐body metabolism and also serves as an important contributor to maintain glucose homeostasis (Shepherd & Kahn 1999). Birds have higher plasma glucose concentrations than other vertebrates of similar body mass in part to the insulin insensitivity for the absence of Glut4 which can decrease blood glucose after feeding in mammals (Polakof *et al*. 2011; Xiong & Lei 2021). Therefore, how to maintain energetic and redox homeostasis in pectoralis would be a great challenge for birds surviving under hypoxic hypothermia.

**Figure 1:**
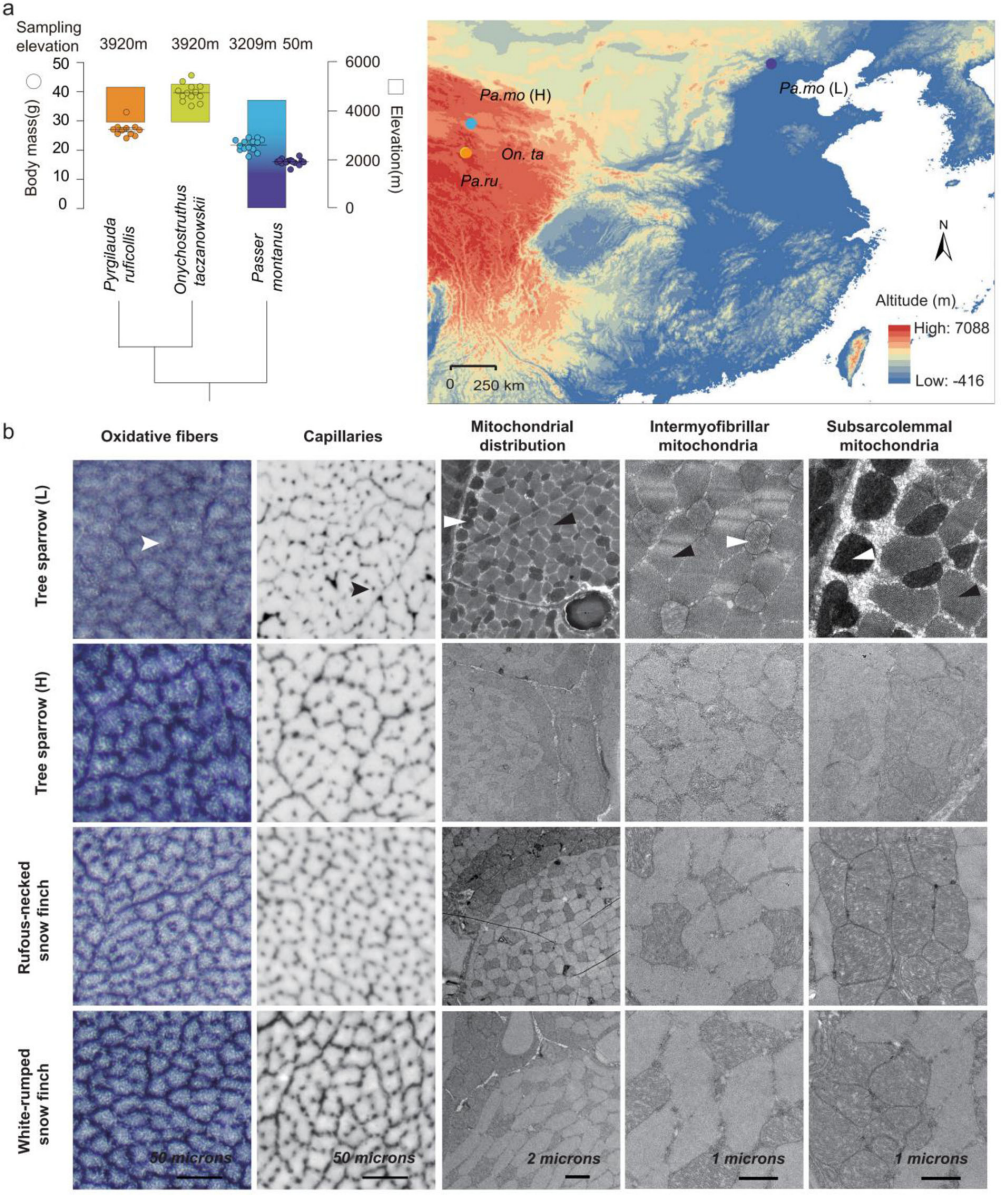
Sampling elevations and body mass of 3 bird species, histological analysis, and electron microscope structure of pectoralis major. (a) Sampling elevations and body mass of 3 species. Phylogenetic tree is reconstructed based on complete mitochondrial genome. Maps were generated using ArcGis (https://www.esri.com). (b) Oxidative fibers and capillaries were identified via staining in succinate dehydrogenase activity and alkaline phosphatase activity, respectively; representative transmission electron micrographs for pectoralis major. White arrow, muscle fiber; black arrow, capillary; white arrowhead, mitochondria; black arrowhead, myofibril.

Snow finches (*Onychostruthus taczanowskii* and *Pyrgilauda ruficollis*) and tree sparrow (*Passer montanus*) are closely related within the Old World sparrows (Passeridae) (Päckert *et al*. 2021). Snow finches are endemic species on the Qinghai−Tibet Plateau (QTP) with a strict elevation distribution range from 3500 to 5100 m, whereas the tree sparrow is an introduced colonizer on the QTP with altitudinal distribution from sea level to 4400 m, which migrated with Tibetans as previous study (Fig. 1a) (del Hoyo *et al*. 2018; Qu *et al*. 2020). Snow finches and high‐altitude tree sparrow separately have relative longer to shorter evolutionary time to the QTP, which comprise a powerful model system to determine the pectoralis variation and its underlying genetic and metabolic basis under the high‐altitude environments.

Based on this model system, we combined phenomic, transcriptomic, and metabolomics measures to the following 3 objectives in this study. First, we found evidences of muscle differences on fiber composition, capillarity, mitochondrial distribution, and abundance across altitudinal songbirds through histochemical methods. Second, we utilized a functional transcriptome approach to decide the regulatory basis of these variations through the differentially expressed genes (DEGs) and co‐expression network analysis (WGCNA). Third, we used highly sensitive gas chromatography–mass spectrometry (GC–MS) combined with the RNA‐seq data to identify their own metabolic mechanisms.

## MATERIALS AND METHODS

### Sampling

Wild birds were caught with mist‐net in summer of 2016, highland bird populations from Qinghai Province about 3200 m (37.033°N, 99.73°E, 10 tree sparrows, *P. montanus*) and 3900 m (35.4°N, 99.25°E, 10 rufous‐necked snow finch, *P. ruficollis* and 10 white‐rumped snow finch, *O. taczanowskii*) and lowland population (40.39°N, 116.67°E, *n* = 8 for transcriptome and 10 for GC–MS analysis) of the tree sparrow from Yanqi Lake of Beijing at 80 m (Table S1, Supporting Information). 100–200 μL of bird blood was collected from wing vein, centrifuged 10 min at 2500 rpm and separately stored at −80°C. Birds were euthanized through heart‐compressed method after blood collection when sampling. Pectoral major muscle was dissected immediately following euthanasia, flash‐frozen in liquid nitrogen, and stored at −80°C. All procedures performed on animal were approved by Institute of Zoology Animal Care Committee.

### Muscle histology and transmission electron microscopy

Oxidative muscle type and capillarity were performed following Scott and Johnston (2012). Briefly, pectoral major muscle was dissected and samples were taken third way along the length of the sternum, covered with OCT, and frozen in isopentane (cooled in liquid N_2_). Muscle sections (10 mm) were obtained in a Cryostat Microtome (Leica CM900, Germany) at −20 ℃. More details were presented in the Supporting Information.

The flight muscle was removed from an intermediate depth and then fixed at 4°C for 24–48 h in 2% glutaraldehyde in 0.1 M PBS buffer at pH 7.4. Small muscle blocks (2 mm × 2 mm) were prepared and post‐fixed in 1% osmium tetroxide in 0.1 M PBS buffer for 1 h, dehydrated in a graded ethanol series (50%, 70%, 70%, 95%, 95%, 100%, 100%), and embedded in epoxy resin. Ultra‐thin sections were cut on a Leica UC7 ultramicrotome and placed on copper grids. The sections were post‐stained with uranyl acetate and lead citrate. Images were collected using a transmission electron microscope (Tecnai G2 F20 TWIN TMP, USA). We measured mitochondrial volume density using stereological methods as previously described (Weibel 1979). Grid size of 90 nm was used to estimate mitochondria and lipid droplet volume at a square size of 4460 × 4460 nm.

### Plasma glucose, lactate, insulin, tissue glycogen measurement, and enzyme activity assays

To quantify circulating glucose levels, fasting plasma was obtained from 8 birds from each species and was measured using an Accu‐Chek blood glucose meter (Roche Diagnostics). Insulin content of fasting plasma was performed using insulin ELISA kit (Cloud‐Clone), lactate in plasma and muscle glycogen were determined with a relative assay kit (Solarbio) according to the manufacturer's instructions. More information about enzyme activity is presented in Supporting Information.

### RNA isolation, library preparation, sequencing, quantification, and normalization

Total RNA was extracted from each pectroralis major muscle of the 38 samples (8 lowland tree sparrows, 10 highland tree sparrows, 10 rufous‐necked snow finches, and 10 white‐rumped snow finches) using Trizol RNA isolation reagents (Invitrogen Corp., Carlsbad, CA). RNA integrity was assessed using the RNA Nano 6000 Assay Kit of the Agilent Bioanalyzer 2100 system (Agilent Technologies, CA, USA). A total amount of 3 μg RNA per sample was used as input material for the RNA sample preparations. Sequencing libraries were generated using NEBNext UltraTM RNA Library Prep Kit for Illumina (NEB, USA) following manufacturer's recommendations and index codes were added to attribute sequences to each sample.

The clustering of the index‐coded samples was performed on a cBot Cluster Generation System using TruSeq PE Cluster Kit v3‐cBot‐HS (Illumia) according to the manufacturer's instructions. After cluster generation, the library preparations were sequenced on an Illumina Hiseq platform and 125 bp/150 bp paired‐end reads were generated. Trimmomatic (Bolger *et al*. 2014) was used to filter reads containing adapter, reads containing ploy‐N and low quality reads based on read quality checked with FASTQC (Andrews 2016). The parameters used were as follows: sliding window = 4‐bp; Phred 33 quality scores = 20; Min read length = 50. Adapter sequences, if detected, were removed. Clean data with high quality was mapped from each species to respective genomes using STAR with default parameters (Dobin *et al*. 2013). We used the reciprocal best‐hit method to generate tree sparrow–rufous‐necked snowfinch and tree sparrow–white‐rumped snowfinch orthologs, respectively. The orthologs shared by 3 species were obtained by intersecting the lists of above 2 orthologs. After the reads were mapped to the reference genomes (Qu *et al*. 2020), expression quantifications of genes and transcripts were performed using RSEM (Li & Dewey 2011). Expression levels for genes with one‐to‐one orthologs in all 3 bird species (*n* = 12 951) were normalized with a RLE (relative log expression) method across muscle samples (Maza 2016).

### Differential expression analysis and weighted gene coexpression network analysis

To identify genes related with pectoralis variation across altitudinal songbirds, we performed differential expression analysis. Differentially expressed genes (DEGs) were calculated based on the Negative Binomial distribution and independent filtering was enabled in a R/Bioconductor package Deseq 2 (R version 3.5.1) with a false discovery rate (FDR) < 0.05 based on Benjamini–Hochberg method to control the FDR in multiple tests context in identifying significantly differentially expressed genes (Love *et al*. 2014). The cut off values for log‐2‐fold change were set at 0.59 and −0.59. Only genes with count number greater than 1 in at least 4 samples were included in differential gene expression analysis.

Then we performed a weighted gene co‐expression network analysis via WGCNA to identify gene modules associated with muscle phenotypic variation and its potentially genetic basis. Only inter‐ and intra‐species differentially expressed genes (DEGs) were included in the analysis. Briefly, we constructed the weighted gene co‐expression network using the normalized, log_2_‐transformed counts to analyze the DEGs with the blockwiseModules function in WGCNA (Langfelder *et al*. 2008) for inter‐ and intra‐species, respectively. An adjacency correlation matrix is calculated for the DEGs, and the correlations are weighted to a soft threshold power β which favors strong correlations over weak one (Zhang & Horvath 2005). For each pair of genes, a robust measure of network interconnectedness is calculated based on the adjacency matrix. For our analysis, the parameters used were as follows: for population network, maximum block size = 966 genes, power (β) = 18; for species network, maximum block size = 2457 genes, power (β) = 22; minimum module size = 25; minimum height for merging modules = 0.25; maximum height for cutting the tree = 0.90. The remaining parameters were kept at the default settings.

Co‐expression modules associated with phenotypic gradients were identified using a principal component analysis (PCA) of gene expression with the blockwiseModules function in WGCNA. Each module was summarized by an eigengene, which is the first principal component of the scaled module expression. Thus, the module eigengene explained the maximum amount of variation of the module expression levels. A Student's asymptotic test with the corPvalueStudent function was used to determine *P* values of the correlation. To specify genes potentially explaining muscle phenotypic variation, we identified hub genes which may be central to the architecture of the regulatory networks represented by each co‐expression module. The hub genes are calculated by their first principal component (PC1), the module eigengene (a summary of overall module expression) (Han *et al*. 2004). We then implemented gene ontology categories within modules positively and negatively correlated with muscle traits using G:PROFILER (FDR < 0.05) (Reimand *et al*. 2007). According to the results of functional GO enrichment analysis, intramodular hub genes were identified as candidate genes driving potentially muscle variation.

### GCMS analysis

Pectoralis (10 lowland tree sparrows, 10 highland tree sparrows, 10 rufous‐necked snow finches, 10 white‐rumped snow finches) and plasma (7 lowland tree sparrows, 7 highland tree sparrows, 6 rufous‐necked snow finches, 7 white‐rumped snow finches) metabolites were extracted by methanol/chloroform protocol, as described previously (Shi *et al*. 2015). More details are presented in Supporting Information.

### Western blot

50 mg of pectoral major muscle from one sample of each species was grinded with liquid nitrogen and then the powder was lysed in 1 mL lysis buffer, 50 mM Tris HCl (pH 7.5), 150 mM NaCl, 5 mM EDTA, 0.5% Nonidet P‐40, and completeMini protease inhibitor cocktail (BC3640; Solarbio). The samples were placed on ice for 4 min and then centrifugated at 12 000 rpm for 15 min at 4°C. 350 μL of the supernatant was transferred to a 2‐mL auto‐sampler vial and was diluted by 3 fold of loading buffer. Protein concentration was quantified using the BCA Protein Assay Kit (PC0020; Solarbio). Supernatant was then separated using SDS‐PAGE for 1 h at 140 V and transferred to PVDF for 2 h at 90 V. Nonspecific antibody bind in was blocked in 5% nonfat dry milk powder in Tris‐buffered saline for 1 h. Blots were incubated in 1% nonfat dry milk powder in Tris‐buffered saline with 0.05% Tween‐20 overnight at 4°C with the following antibodies: rabbit anti‐MEF2C (bs‐4130R; 1:1000; Bioss), rabbit anti‐EPAS1 (bs‐1447R; 1:1000; Bioss), and mouse anti‐β‐actin (bsm‐33036M; 1:1000; Bioss). Blots were incubated with goat anti‐rabbit (bs‐0295G; Bioss) or goat anti‐mouse antibodies (bs‐0296G; 1:3000; Bioss) for 1 h at room temperature. Blots were detected using protein detection reagents (AY0371; Solarbio).

### Phylogenetic analyses

We used MrBayes 3.2 (Ronquist & Huelsenbeck 2003) for the phylogenetic tree construction with the whole mitochondrial genome using best‐fit model (GTR + I + G) based on BIC model selection criteria (Fig. 1a). Sequences were downloaded from GenBank (Accession: NC_024821.1, NC_022815.1, and NC_025914.1 for *P. montanus*, *P. ruficollis*, and *O. taczanowskii*, respectively).

### Statistical analyses

The 2‐tailed student's *t*‐test was used to determine statistical significance. Correlative analysis was carried out using linear regression. For all tables and figures, *P* value < 0.05 was considered significant.

## RESULTS

### Pectoralis phenotypes and PCA

Pectoralis major and body mass of all highland birds were significantly larger than those of lowland tree sparrow, while only highland tree sparrow had a bigger relative ratio of pectoralis major mass to body mass (Fig. 1a and Table 1), reflecting pectoralis as a contributor to the increase of body mass of them. Histological analysis found that highlanders evolved a higher oxidative capacity of pectoralis through increasing the sizes of fiber for highland tree sparrow or myofibril for 2 snow finches (Fig. 1b and Table 1). In contrast to the increased muscle‐fiber size in highland tree sparrows, snow finches had thicker myofibril (Table 1), indicating that different modifications of muscle phenotype occur in intraspecies (high‐altitude tree sparrow vs. low‐altitude tree sparrow) and interspecies (2 snow finches vs. low‐altitude tree sparrow). Additionally, the ratio of wing length to body length significantly associated with muscle fiber area (*R* ^2^ = 0.36, *P* = 0.0002, Fig. S6a, Supporting Information) and myofibril diameter (*R* ^2^ = 0.51, *P* < 0.0001, Fig. S6b, Supporting Information).

**Table 1:** Masses and phenotypes of pectoralis in sparrows

	*Pa. mo* (L)	*Pa. mo* (H)	*Pa. ru*	*On. ta*
Organ mass (mg⋅g^–1^ body mass)^a^				
PMM (g)	1.21 ± 0.04	1.91 ± 0.07^*^	1.73 ± 0.11^*^	1.90 ± 0.10^*^
RM_PM_ (%)	7.50 ± 0.18	8.38 ± 0.30^*^	6.66 ± 0.23^†^	5.45 ± 0.48^†^
Fiber type and capillary density in pectoralis^a^				
FD (mm^–2^)	1723.20 ± 44.79	1373.94 ± 41.74^†^	1887.86 ± 28.06^*^	1768.51 ± 33.67
FS (um^2^)	583.12 ± 15.46	733.09 ± 19.82^*^	530.74 ± 7.77^†^	567.27 ± 10.66
MD (nm)	897.66 ± 41.30	921.90 ± 35.49	1134.71 ± 30.98^*^	1122.51 ± 40.14^*^
CD (mm^–2^)	2869.12 ± 69.68	2506.09 ± 65.23^†^	3731.58 ± 93.67^*^	3409.45 ± 67.62^*^
CA (um^–2^)	8.65 ± 0.69	12.42 ± 1.50^*^	13.26 ± 1.50^*^	13.14 ± 1.86^*^
CF	1.67 ± 0.04	1.84 ± 0.01^*^	1.98 ± 0.03^*^	1.93 ± 0.01^*^
Mitochondrial volume densities and numerical densities^b^				
DS	0.314 ± 0.024	0.481 ± 0.021^*^	0.536 ± 0.007^ ^	0.518 ± 0.013^ ^
Vv (mt)	0.078 ± 0.004	0.092 ± 0.003^ ^	0.114 ± 0.004^ ^	0.118 ± 0.005^ ^
Vv (ssm)	0.047 ± 0.002	0.057 ± 0.001^ ^	0.069 ± 0.002^ ^	0.066 ± 0.002^ ^
Vv (imm)	0.032 ± 0.003	0.034 ± 0.001	0.046 ± 0.003^ ^	0.051 ± 0.004^ ^
Vv (sim)	0.015 ± 0.002	0.023 ± 0.001^ ^	0.023 ± 0.003^ ^	0.015 ± 0.003
Vv (LD)	0.0041 ± 0.0006	0.0060 ± 0.0014	0.0117 ± 0.0017^ ^	0.0133 ± 0.0026^ ^

Data are means ± SE; ^*^ significant increase; ^†^ significant decrease. PMM, pectoralis major mass; RM_PM_, ratio of pectoralis major mass to body mass; FD, fiber density; FS, fiber area; MD, myofibril diameter; CD, capillary density; CA, capillary area; CF, the number of capillaries per fiber; DS, proportion of subsarcolemmal mitochondrion; Vv (mt), total mitochondrial volume density; Vv (ssm), volume density of subsarcolemmal mitochondrion; Vv (imm), volume density of intermyofibrillar mitochondrion; Vv (sim), the difference between Vv (ssm) and Vv (imm); Vv (LD): volume density of lipid droplet. *Pa. mo* (L) (lowland tree sparrow), *Pa. mo* (H) (highland tree sparrow), *Pa. ru* (rufous‐necked snow finch), *On. ta* (white‐rumped snow finch). ^a^ *n* = 8, 10, 10, and 10 for *Pa. mo* (L), *Pa. mo* (H), *Pa. ru*, *On. ta*. ^b^ *n* = 6 for each species.

The improvement in oxidative capacity of flight muscle is also attributed to more capillarity and mitochondrial volume density in highland birds. All highland birds exhibited an enhanced capacity of oxygen transport though the higher capillary to fiber ratio (CF) and capillary size (Fig. 1b and Table 1). Snow finches also had a significantly great capillary density (CD) in comparing to lowland tree sparrows (Table 1). Highland tree sparrow did not increase CD due to a decreased fiber density (Table 1), indicating that increased CF is a compensatory modification for diminishing of fiber density.

Additionally, highland birds had a greater proportion of subsarcolemmal mitochondria (DS) and a greater total mitochondrial volume density (Vv (mt)) in pectoralis (Fig. 1b and Table 1). The last trait in the tree sparrow was entirely contributed to the greater volume density of subsarcolemmal mitochondria (Vv (ssm)), while snow finches had the greater Vv (ssm) and volume density of intermyofibrillar mitochondria (Vv (imm)) (Table 1). Moreover, the Vv (mt) was connected positively with the Vv (LD) (*R* ^2^ = 0.44, *P* = 0.0004, Fig. S6c, Supporting Information) and myofibril size (*R* ^2^ = 0.56, *P* < 0.0001, Fig. S6d, Supporting Information). The difference in mitochondrial abundance between highland and lowland species was greater than the difference between altitudinal populations of the tree sparrow, reflecting that oxidative capacity of flight muscle was greater in snow finches than highland tree sparrows.

The first 3 principal components of the PCA on the basis of 12 phenotypic traits explained most of variations in pectoralis across inter‐ and intra‐species (Fig. S1, Supporting Information). These traits can be grouped into 5 classes including body weight, lipid volume density, muscle fiber or myofibril (FD, FS and MD), capillarity (CD, CA and CF), and mitochondria (Vv (mt), Vv (ssm), Vv (imm), and Vv (sim)). Within species, the PC1 (59.1%) represented mainly most of the variance in mitochondrial volume density, lipid droplet, capillarity, and weight. PC2 (13.1%) and PC3 (8%) were also explained by myofibril and volume density of intermyofibrillar mitochondria. In addition, PC1 (58.7%) among species showed variance of capillarity, myofibril, and mitochondrial morphology, while PC2 (18.4%) and PC3 (6.5%) explained muscle fiber density, capillary size, as well as lipid droplet, respectively. These results showed that the PC1 of PCA could reflect most of muscle phenotype variations.

### Gene expression differences and WGCNA

We sequenced 38 samples of pectroralis major muscle for 2 snow finches and 2 tree sparrow populations, which had the matching muscle phenotype. The G20 and G30 of each species and populations were more than 98% and 90%, and GC content was all around 49–50%. After quality control, we generated a total of 141 Gb of high‐quality RNA‐seq data for subsequent analyses. Samples were aligned to their respective reference genomes, with overall mapping rates ranging from 87.51% to 94.58% across 38 samples. The mean alignment rates were 90.83%, 90.98%, 92.55%, and 91.92% for lowland tree sparrow, highland tree sparrow, rufous‐necked snow finch, and white‐rumped snow finch, respectively. Therefore, our sequencing data was of high quality and was robust for differentially expressed analyses (Table S1, Supporting Information).

Transcriptomic analysis identified 966 differentially expressed genes (DEGs) in highland tree sparrow and 2457 DEGs shared in 2 snow finches comparing to lowland tree sparrow (Table S2, Supporting Information). 407 DEGs, comprising 196 upregulated genes and 211 downregulated genes, appeared to all highland birds. Up‐regulated DEGs were enriched for “muscle structure development” (*P* = 0.0244) and “oxygen transport” (*P* = 0.0078), and “primary bile acid biosynthesis (*P* = 0.0079)” (Table S3, Supporting Information). Down‐regulated DEGs were enriched into “autophagy” (*P* = 0.0147), “apoptotic process” (*P* < 0.0064), and “proteasome” (*P* < 0.0001) (Table S3, Supporting Information).

**Figure 2:**
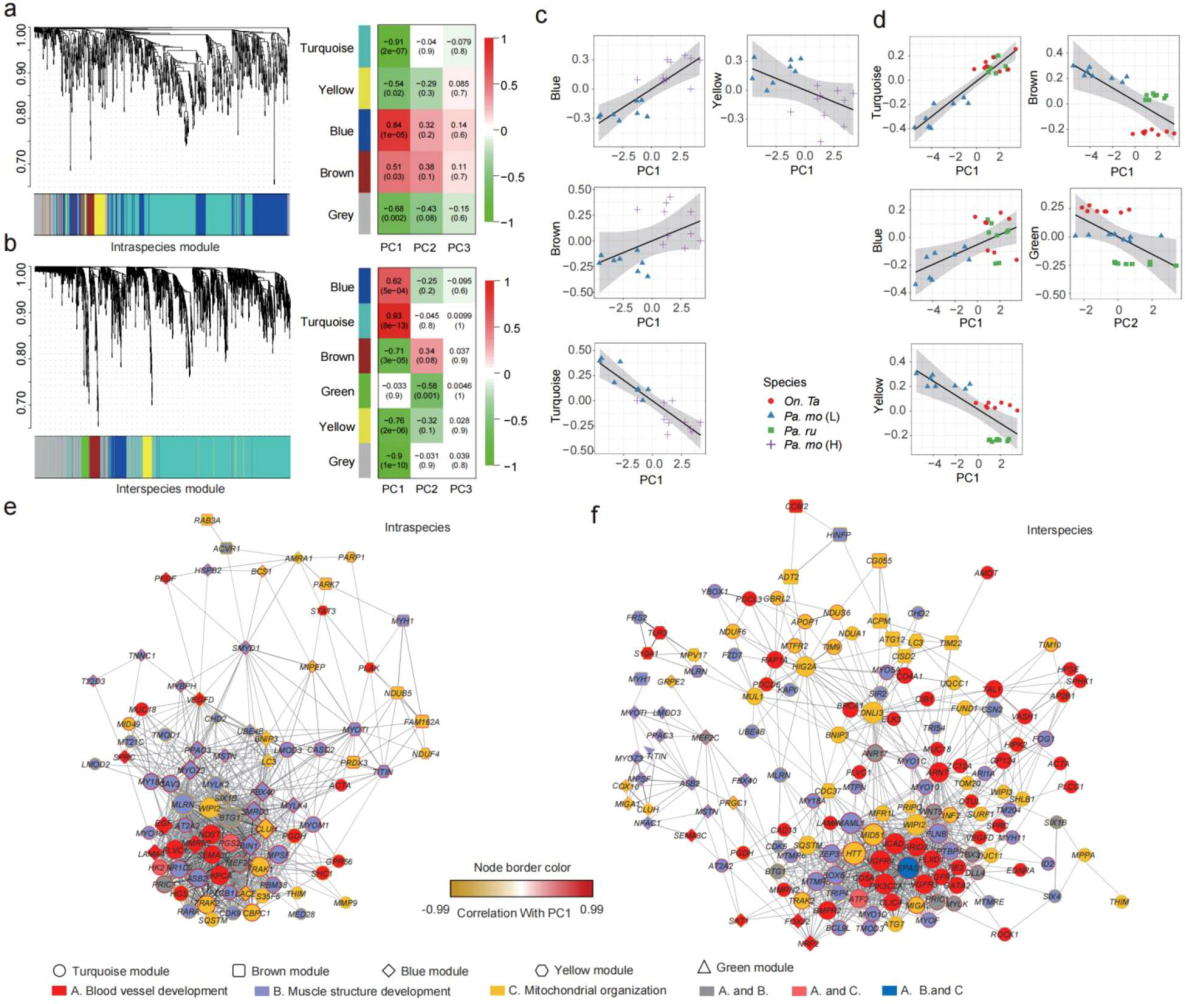
Co‐expression analyses of differentially expressed genes and muscle traits via WGCNA. Average linkage clustering tree and correlation between co‐expression modules and muscle traits across intra‐species (a) as well as inter‐species (b). (c) Co‐expression modules identified via WGCNA. Four modules are associated significantly with PC1 of pectoralis major variation between populations (2 positively connected modules, blue module: 300 genes, brown module: 50 genes; 2 negatively connected modules, yellow module: 51 genes, turquoise module: 438 genes). (d) Four modules are correlated with PC1 of pectoralis major variation across species (2 positively associated module, turquoise module: 1417 genes, blue module: 181 genes; 2 negatively connected modules, brown module: 113 genes, yellow module: 103 genes). Green module (68 genes) is associated negatively with PC2. Network view of angiogenesis, muscle development, and mitochondrial organization in correlated modules in intra‐species (e) and inter‐species (f) level. Shapes indicate modules and are colored according to GO terms. Shape size is presented in accordance with module membership, and the line border color reflects the correlation with PC1. The co‐expression between genes is reflected by edge line width.

Weighted gene correlation network analysis using WGCNA organized 966 DEGs within populations and 2457 shared DEGs in snow finches to 4 and 5 modules, respectively (Fig. 2a,b; Figs S2–S4, Supporting Information). In population network (high‐ and low‐altitude tree sparrows), blue module and brown module correlated positively with PC1 (Fig. 2c). The DEGs in blue module were enriched into “muscle structure development” (*P <* 0.001) and “muscle contraction” (*P* = 0.0281) (Table S4, Supporting Information). The DEGs in brown module were enriched into “mitochondrion” (*P <* 0.001), “respiratory chain” (*P <* 0.001) and “oxidative phosphorylation” pathway (*P* = 0.001) (Table S4, Supporting Information). Also, yellow module and turquoise module associated negatively with PC1 (Fig. 2b). The DEGs in these modules were separately enriched into “organelle subcompartment” (*P* = 0.0300) and “muscle system process” (*P* = 0.001), “mitochondrion” (*P* = 0.023) as well as some metabolic pathways (e.g. “carbohydrate derivative metabolic process” [*P* < 0.001] and “citrate cycle” [*P* = 0.037]) (Table S4, Supporting Information). As to species network, blue module and turquoise module associated positively with PC1 (Fig. 2d). The DEGs in turquoise module were enriched into many developmental processes including “muscle structure development” (*P* < 0.001) and “vasculature development” (*P* < 0.001), “mitochondrion” (*P* < 0.001), and some metabolic processes such as “lipid metabolic process” (*P* = 0.009) and “inositol phosphate metabolism” (*P* = 0.001) (Table S4, Supporting Information), and yellow module and brown module associated negatively with PC1 (Fig. 2d). The DEGs in these modules were respectively enriched into “organelle” (*P* = 0.009) and “membrane‐bounded organelle” (*P* < 0.001) (Table S4, Supporting Information). Only green module had a negative correlation with PC2 (Fig. 2b) and genes in this module were related to “membrane part” (*P* = 0.004) (Table S4, Supporting Information). These gene ontology (GO) enrichment and KEGG pathways associated with angiogenesis, muscle development, mitochondrial organization, and metabolic process, suggested the potential regulatory basis of muscle phenotypes.

### Discovery of candidate genes underlying phenotypic variations

**Figure 3:**
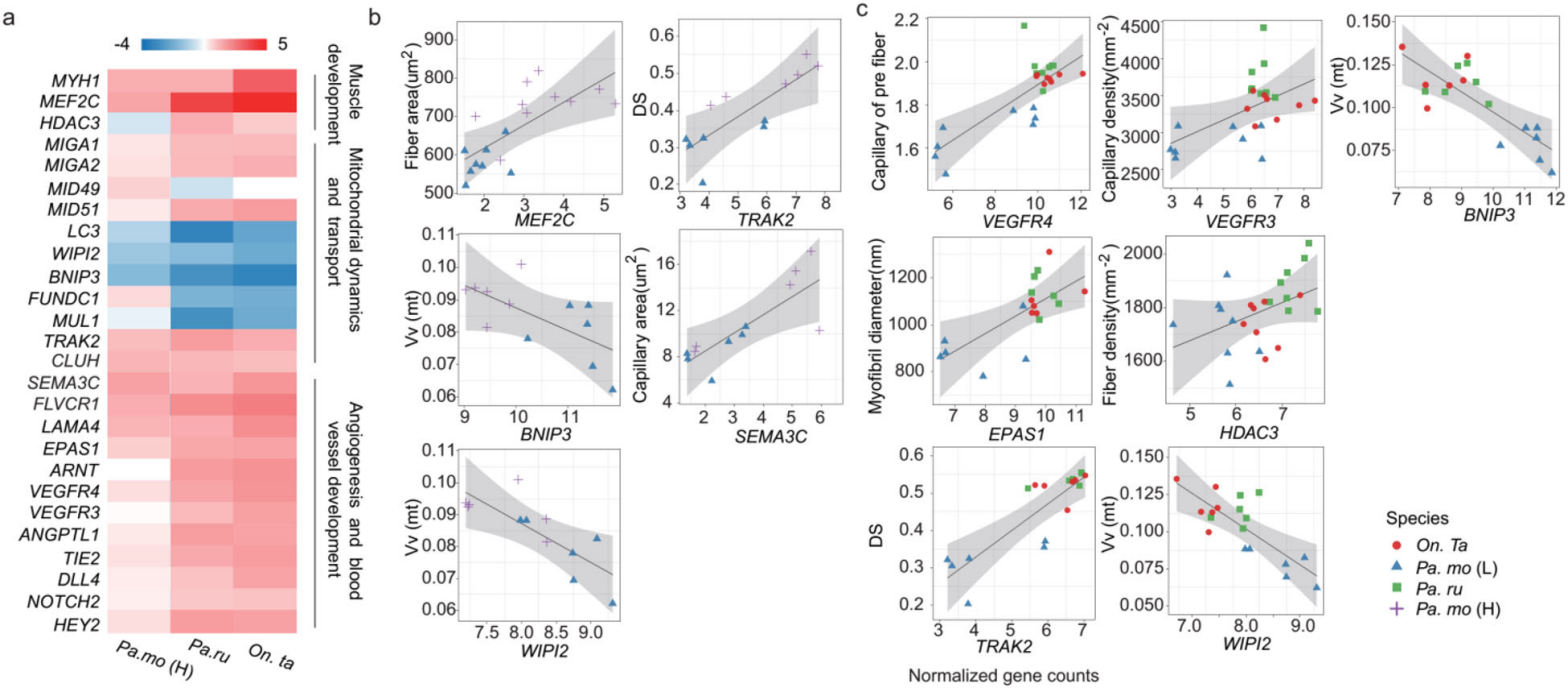
Potential genetic basis of muscle development, angiogenesis, and mitochondrial organization. (a) Gene expression changes (log_2_fold change) associated with muscle structure development, angiogenesis, and mitochondrial organization in highland birds relative to the lowland tree sparrow. Associations between hub gene expression and several muscle traits in populations (b) and among species (c). Statistically significant associations (linear regression, *P* < 0.05) were reported.

The hub genes in co‐expression networks are considered having the potential biological relevance and importance in driving phenotypic variations, so we identified hub genes within each module by connectivity and degree (top 30% and 15% of genes as hub genes in population and species network, respectively) (Table S5, Supporting Information). As to population network, 9 hub genes in blue module involved in blood vessel formation (*VEGFD, EGF, MMRN2*), muscle cell differentiation (*MYOZ3, PPAC3, SMRD3, FBX40*), mitochondrial distribution (*CLUH*), and mitophagy (*BNIP3*) (Figs 2e,3a; Table S5, Supporting Information). And 8 genes were shared DEGs with snow finches, pointing to a common genetic basis of muscle phenotype. Of these genes, *CLUH* positively associated with DS within altitudinal populations (*R* ^2^ = 0.87, *P* < 0.0001) but not species (*R* ^2^ = 0.15, *P* = 0.1102) (Table S6, Supporting Information). *BNIP3* acted as a pro‐apoptotic factor combining with autophagosome (Ni *et al*. 2015), and its expression connected negatively with the Vv (mt) within altitudinal populations (*R* ^2^ = 0.41, *P* < 0.024) and species (*R* ^2^ = 0.65, *P* < 0.0001) (Fig. 3b; Table S6, Supporting Information). Eleven hub genes in turquoise module were involved in blood vessel development (*PGS2, KPCA, NDST1, FLVC1, SEMA3C*), muscle system process (*MEF2C, BTG1, PRIC1*), and mitochondrion localization (*TRAK1, WIPI2, TRAK2*) (Figs 2e,3a; Table S5, Supporting Information). *SEMA3C* affected endothelial cell proliferation through increasing integrin activity similar to those induced by VEGF (Banu *et al*. 2006), and its expression correlated positively with capillary area within altitudinal populations (*R* ^2^ = 0.64, *P* = 0.002) (Fig. 3b; Table S6, Supporting Information). *MEF2C* was related to striated muscle hypertrophy and obviously connected with fiber area within altitudinal populations (Fig. 3b; Table S6, Supporting Information). *TRAK2* enhanced mitochondrial transport in axons of hippocampal neurons (MacAskill & Kittler 2010), and demonstrated a positive correlation with the DS within altitudinal populations (*R* ^2^ = 0.67, *P* = 0.001) and species (*R* ^2^ = 0.73, *P* < 0.0001) (Fig. 3b; Table S6, Supporting Information). Additionally, *WIPI2* was suggested a mitophagy factor (Ni *et al*. 2015), and its expression showed a negative association with the Vv (mt) (Fig. 3b; Table S6, Supporting Information).

As to species network, 2 hub genes of the blue module were involved in vasculature development (*MEF2C*), and muscle structure development (*MEF2C, ASB2*) (Figs 2f,3c; Table S5, Supporting Information). The turquoise module had 12 hub genes, 11 of these (expect for *WIPI2*) only in snow finches but not in highland tree sparrow comparing with lowlander, probably indicating the regulatory difference of pectoralis phenotypes between snow finches and highland tree sparrow. The 5 genes in this 12 hub gene subset were involved in angiogenesis (*EPAS1, VEGFR3, VEGFR4, PIK3C2A, KIAA1462*), 3 were involved in muscle structure development (*FLNB, MAML1, EPAS1*), and 6 were involved in mitochondrion organization (*LIG3, HIGD2A, WIPI2, EPAS1, MID51, HTT*) (Figs 2f,3c; Table S5, Supporting Information). *EPAS1*, comprising a dimeric complex with *ARNT* responding to hypoxia, was upregulated in snow finches and played a central role on development process including angiogenesis, muscle development, and mitochondrion organization in species network (Fig. 2f; Table S6, Supporting Information) (Rankin *et al*. 2008; Skuli *et al*. 2009; Park *et al*. 2016). In addition, several key downstream genes of *EPSA1* including *VEGFR3*, and *VEGFR4*, mediating angiogenesis as well blood vessel morphogenesis, were also overexpressed hub genes and associated with CD (*VEGFR3*, *R* ^2^ = 0.28, *P* = 0.0074) and CF (*VEGFR4*, *R* ^2^ = 0.62, *P* < 0.0001) across species (Fig. 3c; Table S6, Supporting Information). *MID51* involving in mitochondrial fission was a hub gene with a higher degree and its expression positively associated with the Vv (mt) (*R* ^2^ = 0.74, *P* < 0.0001). In these modules, some genes would have not high intramodular connectivity and degree via WGCNA, while function enrichment analyses and association test with muscle phenotypes also revealed an essential impact on regulating skeletal muscle development, angiogenesis, and mitochondrial biogenesis (Figs 2f,3a; Table S6, Supporting Information).

To confirm the real expression of some hub DEGs from protein level, we performed western blots for 2 key genes including *MEFC* and *EPAS1*, for an example. In accord with normalized transcript counts, protein levels of *MEF2C* were improved in all highland songbirds compared to lowlanders, while *EPAS1* only in rufous‐necked snow finch and white‐rumped snow finch but not highland tree sparrows (Fig. S7, Supporting Information) (Xiong *et al*. 2020).

### Metabolic basis of phenotypic variations

**Figure 4:**
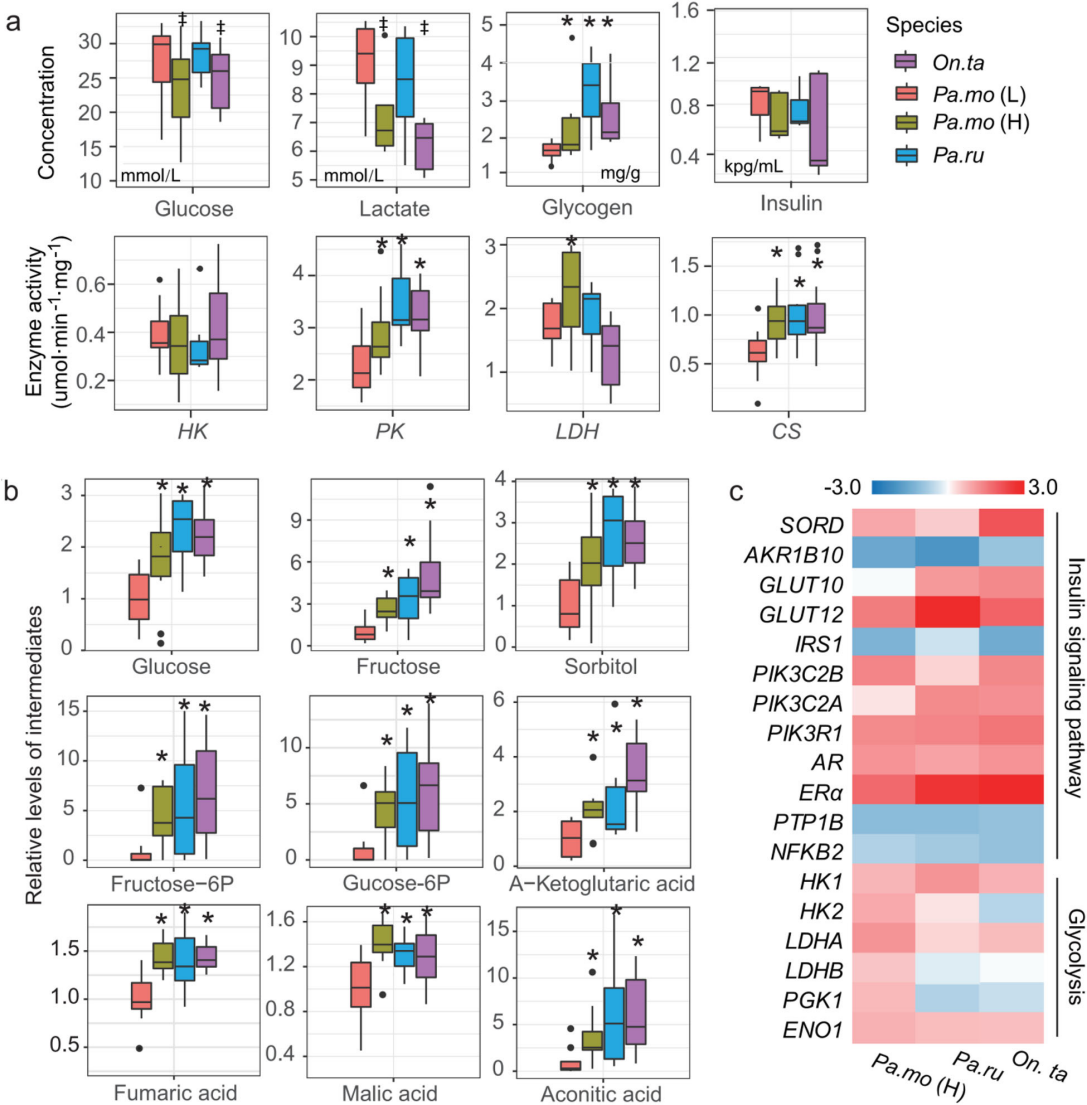
Increase in capacities of insulin sensitivity, glycolysis, and TCA cycle. (a) Glucose, lactate, and insulin concentration in plasma, glycogen content, and enzyme activity difference involving in glycolysis and TCA cycle in pectoralis. * significant increase; † significant decrease. (b) Intermediate concentrations of glycolysis and TCA cycle. * significant increase; † significant decrease. (c) Gene abundance involving in the increase of glycolysis, insulin sensitivity, and polyol pathway.

Given that skeletal muscle plays a central role on whole‐body metabolism, we used highly sensitive GC–MS combined with the RNA‐seq data to identify metabolic features in different muscle phenotypes. Fasting plasma glucose was lower in most of highland birds, while lots of monosaccharides and glycogen were accumulated in muscle fiber (Fig. 4a,b; Fig. S5a, Supporting Information), indicating improvement in glucose uptake and utilization. Highland songbirds including white‐rumped snow finch and tree sparrow have significant low plasma lactate despite rufous‐necked snow finch slightly decrease lactate (Fig. 4a). Although plasma insulin concentration was similar to those of lowland birds (Fig. 4a), highland birds had some DEGs involved in regulating insulin sensitivity and insulin signaling pathway (*AR/ERα, PIK3C2A*, *PIK3C2B*, *SLC2A12* [Glut12], and *PTP1B*) (Fig. 4c). Additionally, expression and activities of some glycolytic enzymes (hexokinase, pyruvate kinase, and lactate dehydrogenase) were increased in highland birds (Fig. 4a,c). Consistently, glycolytic intermediates increased in all highland birds, with increased glucose and fructose‐6‐phosphate (Fig. 4b). Activity of citrate synthase (CS) was higher in highland birds (Fig. 4a), which supported a great Vv (mt) in pectoralis (Table 1). Meanwhile, concentrations of 6‐, 5‐, and 4‐carbon intermediates downstream of CS (aconitate, α‐ketoglutarate, fumarate, and malate) were increased (Fig. 4b). Collectively, the improvement of insulin sensitivity and glucose utilization was a shared feature of muscle metabolism to hypoxia through activating insulin signaling pathway.

**Figure 5:**
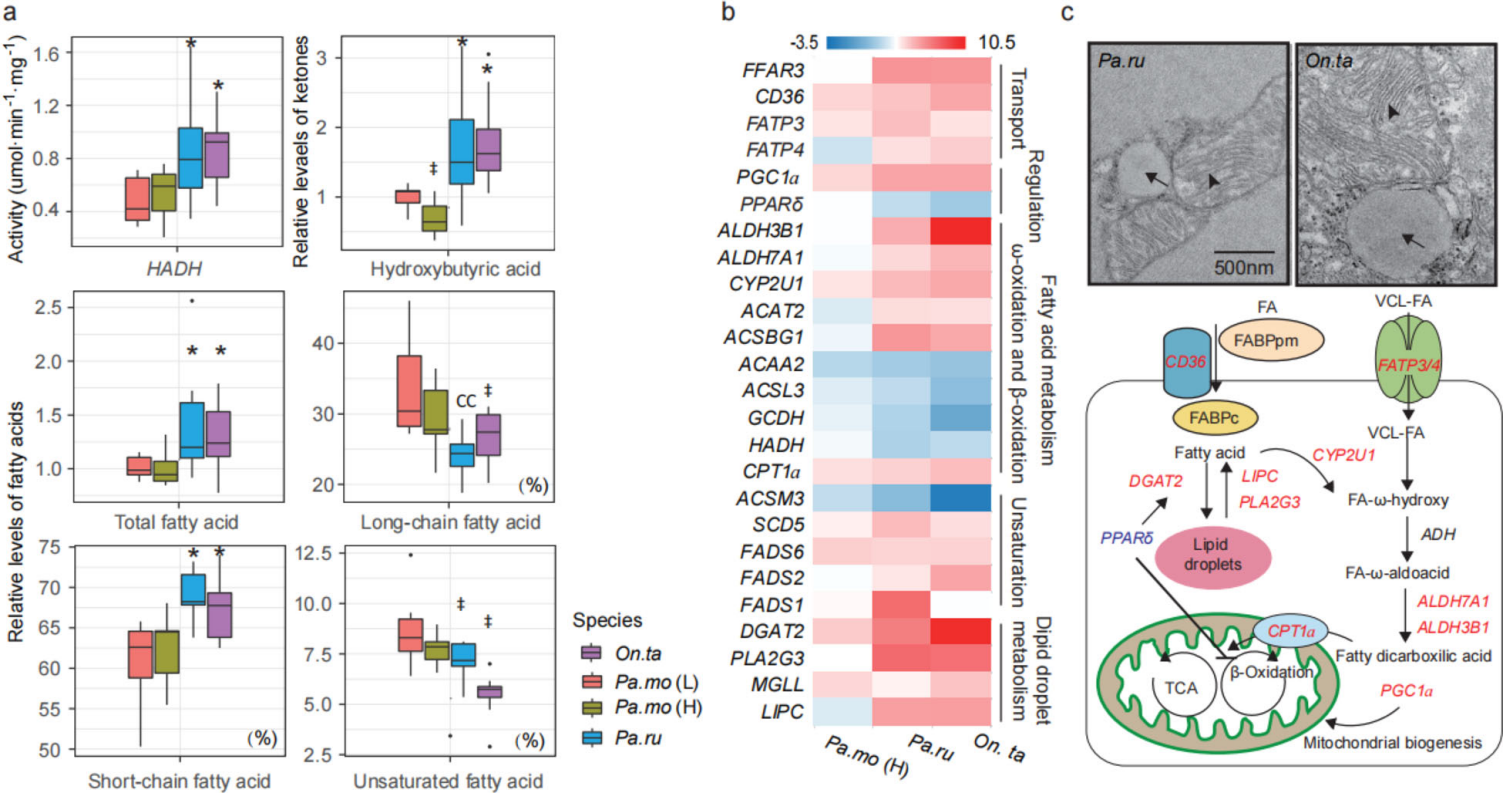
Fatty acid biosynthesis and oxidation in pectoralis. (a) 3‐hydroxyacyl‐CoA dehydrogenase (*Hadh*) activity, hydroxybutyric acid content, total fatty acid content, long chain/total fatty acid ratio, short chain/total fatty acid ratio, and unsaturated/total fatty acid ratio in pectoralis. * significant increase; † significant decrease. (b) Expression of genes for fatty acid transport, ω‐oxidation, fatty acid degradation, and regulation of fatty acid metabolism were differentially expressed in snow finches. (c) Schematic summary of fatty acid metabolism in pectoralis. Black arrow, lipid droplet; black arrowhead, mitochondria.

Muscle phenotype suggested that snow finches had greater capacities of oxygen transport and mitochondrial oxidation, which probably predicted alternative fuel substrates in snowfinches. Consistently, muscle metabolite levels also indicated that total fatty acid concentration increased in 2 snow finches (Fig. 5a; Fig. S5b, Supporting Information). The ratio of short‐chain fatty acid (SFA), however, was great in snow finches instead of a low ratio of desaturated fatty acid (DFA) (Fig. 5a). Meanwhile, the ratio of long‐chain fatty acid (LFA) also significantly decreased in snow finches (Fig. 5a), indicating DFA and LFA might be important fuel substrates for thermogenesis under low temperature. Additionally, *PPARδ* had a low expression level in snow finches, while *DGAT2* activating lipid depot formation was up‐regulated and its expression was positively associated with volume density of lipid droplet (*R* ^2^ = 0.49, *P* = 0.0002) (Fig. 5b; Table S6, Supporting Information). Interestingly, *PGC1a* and some PPAR target genes, involving in fatty acid transport, ω‐oxidation, and lipid droplet degradation, had an increased expression and activity of *HADH* was also dominantly increased (Fig. 5a–c). Together, snow finches likely decrease *PPARδ* expression to promote intramuscular lipid biosynthesis and increase mitochondrial metabolism for fatty acid oxidation for suffering a long highland life history. In contrast, hydroxybutyrate (HB) decreased significantly in highland tree sparrow (Fig. 5a), indicating that ketone instead of fatty acid was a major anaplerotic substrate for energetic supply in a short evolutionary time to hypoxia.

## DISCUSSION

By integrating a comprehensive array of analytical techniques, we illuminate muscle phenotypic variation and its underpinning regulatory as well as metabolic mechanism across altitudinal songbirds with different highland lifetime history. Despite considerable increases in muscle oxidative capacity, capillarity and mitochondrial abundance when facing hypothermia and hypoxia, different regulatory mechanisms might drive their occurrence between snow finches and high‐altitude tree sparrow. A metabolic feature shared by all highland birds is the improvement in insulin sensitivity and glucose utilization through activating insulin signaling pathway, which is vital to maintain metabolic homeostasis in highland birds. Additionally, an alternative fuel would be taken by highland birds with different lifetime under hypothermia environment.

### Pectoralis variations for highland survival

Great body mass and muscle weight in highland animals are in concordance with Bergmann's rule (Sun *et al*. 2017; Fan *et al*. 2019). Avian pectoralis is used to support the high metabolic costs through aerobic metabolism for flight or thermogenesis (Marquez *et al*. 2006). High‐altitude birds, however, suffer from severely limited oxygen supplement due to decrease in the partial pressure of oxygen. Therefore, the physiological responses to hypoxic hypothermia of highland are generally found by increasing oxidative capacity as well as oxygen delivery and by altering oxygen utilization in pectoralis (Scott *et al*. 2009; Xiong *et al*. 2020). Either size modification of muscle fiber or myofibril in highland birds plays an important role in heat production through shivering thermogenesis for maintenance of a constant core body temperature (Chaillou 2018). Additionally, various modifications of muscle phenotype across altitudinal birds are probably associated with the flight performance in hypoxic environment. For example, an increased size of myofibril would produce more contractile force to prevent flightless, probably accounting for why highland tree sparrows perform a weak flight capacity (Sun *et al*. 2016).

The improvement in oxidative capacity of pectoralis also attributes to more capillarity and mitochondrial content in highland birds. The increases in CD and CF are thought to enhance the diffusion capacity of oxygen from blood to muscle (Scott & Milsom 2007; Xiong *et al*. 2020). Capillary size increase may reduce capillary flow resistance directly and maintain capillary rheology in hypoxic environment (Hauck *et al*. 2004). Mitochondrion is the main place of aerobic oxidation and takes a majority of ATP production in eukaryote (Rich 2003). An increase in DS in highland birds may enhance ability to produce proton‐motive force as reported in previous studies (Mahalingam *et al*. 2017). Overall, capacities of oxidation and oxygen delivery in pectoralis are prompted through differentially phenotypic variations in highland tree sparrow and snow finches, probably resorting from different adaptation time to hypoxic hypothermia.

### Potential basis of pectoralis variations in differential gene expression

Given that the differences in muscle fiber and myofibril occur in highland tree sparrow and snow finches comparing to lowland tree sparrows, respectively, we propose the potential regulatory basis of pectoralis variation through DEGs and WGCNA analyses. Some genes are found to be involved in the regulation of muscle development via *MRF4‐MEF2* axis, which is thought to be a novel pathway regulating muscle fiber size and muscle mass in adult muscle tissue (Moretti *et al*. 2016). *MRF4* and *MEF2C* are known to play a role in myogenesis (Hernandez‐Hernandez *et al*. 2017). *MRF4* knockdown in skeletal muscle of adult rodent stimulates *MEF2* transcriptional activity and then causes muscle hypertrophy (Moretti *et al*. 2016). However, *HDAC3*, which can deacetylate and repress *MEF2C*, can be up‐regulated by hypoxia‐induced factor (*HIF‐1α*), and increases cardiomyocyte hyperplasia in mice (Wu *et al*. 2011). Therefore, *MRF4‐MEF2* axis likely controls muscle hypertrophy in highland tree sparrow and causes muscle hyperplasia under overexpression of *HDAC3* induced by *EPAS1* in snow finches, which may also be a regulative process of postnatal but not embryonic myogenesis (Moretti *et al*. 2016).

All highland birds have an increase in capillary size along with the overexpression of *SEMA3C* which is also a hub gene in the highland tree sparrow. Therefore, *SEMA3C* probably increases integrin activation to control blood vessel size in highland birds as previous study proposed (Banu *et al*. 2006). Additionally, snow finches also have an increase in capillary density along with the overexpression of *EPAS1* and its downstream genes. Mounting evidence suggests that *EPAS1* has an important role in angiogenesis, though most of the studies are focused on *HIF1α* (Rankin *et al*. 2008; Skuli *et al*. 2009; Park *et al*. 2016). Overexpression of *EPAS1* and *ARNT* in pectoralis of snow finches probably maintains an enhanced capillarity via controlling expression of target genes both in *VEGF/VEGFR* and *ANG/TIE* pathways (Rankin *et al*. 2008; Skuli *et al*. 2009; Park *et al*. 2016). Importantly, the increased expression of *VEGFR3* promotes the expression of *DLL4* and *HEY2*, and then controls the conversion of tip cell to stalk cell by reinforcing Notch signaling during angiogenic sprouting (Hellstrom *et al*. 2007). Nevertheless, the up‐regulated expression of *TIE2* and *ANGPTL1* stimulates basement membrane deposition and pericyte detachment, thereby mediating vessel maturation (Augustin *et al*. 2009).

Autophagy is a lysosome‐mediated degradation process for cytoplasmic components, and also plays an important role in mitochondrial homeostasis (Lee *et al*. 2012). Highland birds decrease the expression of genes involved in mitophagy, including *WIPI2*, *LC3*, and *BNIP3* (Fig. 3a), indicating a common central role in maintaining mitochondrial volume density. Also, *TRAK2* and *CLUH* are shared regulation genes of mitochondrial distribution in highland birds (MacAskill & Kittler 2010). However, differentially expressed genes for mitochondrial fission and fusion are various. Snow finches and highland tree sparrow induce mitochondrial fission *via MID51* and *MID49*, respectively (Ni *et al*. 2015). Additionally, *MIGA1* and *MIGA2* also enhance mitochondrial fusion only in snow finches (Zhang *et al*. 2016). Besides, *KLF4/ERR/PGC1a* transcriptional complex regulates a broad spectrum of genes involved in mitochondrial biogenesis, dynamics, and metabolism (Dorn *et al*. 2015). All 3 genes are over‐expressed in snow finches, but only *ERRΓ* has an improved expression in the highland tree sparrow, probably indicating an increase in Vv (ssm).

### Insulin sensitivity, glucose utilization, and fatty acid metabolism

One of the prevailing views considers energetic needs for thermogenesis in highland coming from glucose oxidation which requires less O_2_ than fatty acid oxidation (Schippers *et al*. 2012). Glucose uptake from the bloodstream into cells is mediated by a family of facilitative glucose transporters (Gluts) (Shepherd & Kahn 1999; Xiong & Lei 2021). It has been long suggested that a low sensitivity to insulin is due to the absence of Glut4 in birds (Polakof *et al*. 2011). However, recent studies elucidate that Glut12 overexpression parallels that of Glut4, improves insulin sensitivity, and enhances glucose uptake in skeletal muscles of rodent and chicken (Coudert *et al*. 2015; Xiong & Lei 2021). Highland birds exhibit a similar concentration of plasma insulin and an accelerated glucose uptake in flight muscle along with high expression of Glut12, indicating an improvement of insulin sensitivity. Low plasma lactate in highland songbirds was likely driven by high expressions of lactate dehydrogenase (LDH) and also by high activity of LDH in highland tree sparrow. Previous studies have suggested that *AR/ERα*, *PIK3C2A*, *PIK3C2B*, and *PTP1B* mediate insulin sensitivity through activating insulin signaling pathway (Yu *et al*. 2013). It is the first time to find an enhanced glucose metabolism via activating insulin signaling pathway by gene expression in highland vertebrates. Additionally, increase in insulin sensitivity improves glucose oxidation (Yan 2018), which mainly exhibits in increased capacities of glycolysis and TCA cycle (Horscroft *et al*. 2017).

Snow finches also improve the capacities of fatty acid biosynthesis and oxidation, differing from ketone metabolism of the highland tree sparrow. Per mole of fatty acid oxidation can yield more ATPs than glucose despite more oxygen consumption (Schippers *et al*. 2012); thereby, it is an important energy strategy taken by small animals to cold exposure (Cheviron *et al*. 2012). The enhanced capacities of lipid oxidation probably occurs in snow finches facing prolonged cold and originates from the improvement of β‐oxidation as well as ω‐oxidation through overexpression of target genes or higher enzyme activity, which is likely regulated by *PGC1a* (Cheviron *et al*. 2012; Horscroft *et al*. 2017).

## CONCLUSIONS

Determining the molecular and functional mechanisms underlying phenotypic variation is a fundamental goal of evolutionary biology and ecology. In this study, we confirm that highland birds have an extensive increase in capillarity, mitochondrial abundance, and oxidative capacity of muscle fiber. RNA‐seq analyses reveal differentially expressed genes in modules associated with phenotypes enriched in blood vessel, muscle structure development, and mitochondrial organization. Despite similar phenotypes and functional enrichments across highland birds, different molecular and biochemical mechanism drive their occurrence in part due to various evolutionary histories. Moreover, a shared metabolic feature among highland birds is the improvement in insulin sensitivity and glucose utilization through activating insulin signaling pathway, which is vital to maintaining metabolic homeostasis in highland birds. However, fatty acid biosynthesis and oxidation are enhanced in snow finches with a long evolutionary history, also differing from ketone body metabolism in recently introduced colonizer. To our knowledge, this study is the first to investigate pectoralis variation and its regulatory as well as metabolic mechanisms across altitudinal songbirds.

## CONFLICT OF INTEREST

The authors of this manuscript have declared no competing interests.

## Supporting information


**Figure S1** Rotated principal component analysis of flight muscle.
**Figure S2** 18 and 22 of soft power for scalefree network construction of intraspecies (a) and interspecies (b) respectively.
**Figure S3** Modules correspond to muscle samples within species (a) and between species (b).
**Figure S4** Network heatmaps of the 966 and 2457 genes differentially expressed between intraspecies (a) and interspecies (b) respectively.
**Figure S5** Relative levels of intermediates and fatty acids in fasting plasma. * significant increase; ‡ significant decrease.
**Figure S6** Associations between wing length/body length and fiber area (a), between wing length/body length and myofibril diameter (b), between Vv (mt) and Vv (LD) (c), between Vv (mt) and myofibril diameter (d), and between Vv (LD) and myofibril diameter (e).
**Figure S7** Protein levels of *MEF2C* and *EPAS1*.
**Table S1** Specimen information used for phenomic, transcriptomic and metabolomic analyses
Table S2
**Table S3** Gene ontology terms, Human Phenotype Ontology, and KEEG pathways enriched in shared differentially expressed genes
**Table S4** Gene ontology terms, Human Phenotype Ontology, and KEEG pathways enriched in muscle functional module of differentially expressed genes
Table S5
**Table S6** Candidate DEGs connected with phenotypic variations
