# Supplementary material for: Comparative transcriptomic and metabolomic analysis reveals pectoralis highland adaptation across altitudinal songbirds
Source: Integr Zool. 2022 Jan 10;17(6):1162–78. doi: 10.1111/1749-4877.12620 (PMC9786770; doi:10.1111/1749-4877.12620)
Supplement: Supplementary file 1 — Figure S1 Rotated principal component analysis of flight muscle. Figure S2 18 and 22 of soft power for scalefree network construction of intraspecies (a) and interspecies (b) respectively. Figure S3 Modules correspond to muscle samples within species (a) and between species (b). Figure S4 Network heatmaps of the 966 and 2457 genes differentially expressed between intraspecies (a) and interspecies (b) respectively. Figure S5 Relative levels of intermediates and fatty acids in fasting plasma. * significant increase; ‡ significant decrease. Figure S6 Associations between wing length/body length and fiber area (a), between wing length/body length and myofibril diameter (b), between Vv (mt) and Vv (LD) (c), between Vv (mt) and myofibril diameter (d), and between Vv (LD) and myofibril diameter (e). Figure S7 Protein levels of MEF2C and EPAS1. Table S1 Specimen information used for phenomic, transcriptomic and metabolomic analyses Table S2 Table S3 Gene ontology terms, Human Phenotype Ontology, and KEEG pathways enriched in shared differentially expressed genes Table S4 Gene ontology terms, Human Phenotype Ontology, and KEEG pathways enriched in muscle functional module of differentially expressed genes Table S5 Table S6 Candidate DEGs connected with phenotypic variations [file INZ2-17-1162-s003.doc]

**SUPPLEMENTARY MATERIALS**

**Supplementary text**

**Muscle histology and transmission electron microscopy**

Oxidative muscle ﬁbers were identiﬁed by succinate dehydrogenase activity, by staining in assay buffer (concentrations in mM: 0.6 nitroblue tetrazolium, 2.0 KH_2_PO_4_, 15.4 Na_2_HPO_4_, 16.7 sodium succinate) for 1 h at room temperature. Using alkaline phosphatase activity identified muscle capillaries, also by staining for 1 h at room temperature (assay buffer concentrations in mM: 1.0 nitroblue tetrazolium, 0.5 5-bromo-4-chloro-3-in-doxyl phosphate, 28 NaBO_2_,7 MgSO_4_; pH 9.4). The sections were imaged using light microscopy and sufﬁcient images (eight or more) were analysed for each sample using image J software. Biochemicals were obtained from Sigma-Aldrich (Shanghai, China).

**Plasma glucose, lactate, insulin, tissue glycogen measurement, and enzyme activity assays**

The pectoralis was homogenized in 10 volumes of ice-cold homogenization buffer (in mM: 50 hepes, 5 EDTA, 0.2 dithiothreitol (DTT), and 0.1% Triton-X-100; pH 7.4). After homogenates were centrifuged at 1,000 g at 4 ℃, the supernatants were collected and determined for protein contents using the G-250 method. The maximal activities of 5 enzymes (hexokinase, pyruvate kinase, lactate dehydrogenase, 3-hydroxyacyl-CoA dehydrogenase, and citrate synthase) in pectoralis were measured at sparrow rectal temperature (40 ℃) in 100 mM L^-1^ KH_2_PO_4_ (pH 7.4) with a 96-well plate ELIASA. All assays were optimized to assure substrates and enzymes were not limited and carried out in triplicate with the following reaction conditions (in mM, unless stated otherwise).

For carbohydrate metabolism: (i) hexokinase (HK): 10 mM L^-1^ glucose, 3 mM L^-1^ ATP, 10 mM L^-1^ MgCl_2_, 1.5 mM L^-1^ NADP^+^, one unit of glucose-6-phosphate dehydrogenase; (ii) pyruvate kinase (PK): 10 mM L^-1^ phosphoenol pyruvate, 2.5 mM L^-1^ ADP, 10 mM L^-1^ MgCl_2_, 0.15 mM L^-1^ NADH, one unit of lactate dehydrogenase; (iii) lactate dehydrogenase (LDH): 5 mM L^-1^ pyruvate, 0.15 mM L^-1^ NADH. For fatty acid metabolism: (i) 3-hydroxyacyl-CoA dehydrogenase (HOAD): 0.1 mM L^-1^ acetoacetyl CoA, 0.5 mM L^-1^ NADH, 0.2 mM L^-1^ DTT. For the tricarboxylic acid cycle: (i) citrate synthase (CS): 0.5 mM L^-1^ oxaloacetate, 0.15 mM L^-1^ acetyl-coA, 0.15 mM L^-1^ 5,5′- dithiobis-2-nitrobenzoic acid, 0.1% Triton X-100, in 100 Tris (pH 8.0). Background rates were calculated in control reactions lacking specific substrates. Extinction coefficients were 6.22 (mM L^-1^)^−1^cm^−1^ for NADH (340 nm) and 13.6 (mM L^-1^)^−1^cm^−1^ for DTNB (412 nm) to measure enzyme activities.

**Gas chromatography-mass spectrometry analysis**

1 mL of chloroform/methanol/ distilled water mixture (chloroform: methanol: distilled water = 1:4:1 mixture) was added to Eppendorf tubes containing ∼50 mg pectoralis powdered in lipid nitrogen. 450 μL of methanol/distilled water mixture (8:1 mixture) was added to Eppendorf tubes containing ∼50 uL plasma. 20 μg, and 10 μg of heptadecanoic acid as well as decanoic acid as double internal standard were added into muscle and plasma tube, respectively. After vortex for 10 s, samples were kept on ice for 15 min and then in a sonication bath for 15 min. After centrifugation at 12,000 rpm for 15 min at 4 °C, 200 μL of supernatant was transferred to a 2 mL auto-sampler vial and dried in a vacuum oven. Dried samples were derivatized using methoxyamine hydrochloride solution (50 μL of 15mg·mL^−1^ methoxyamine in pyridine). Themixture was kept for 16 h at room temperature for methoxymation, and then 50 μL of TMSFA containing 1% TMCS was added for trimethylsilylated. After 1 h trimethysilylation, 30 μL of hexane was added and then transferred to an insert in 2 mL autosampler vial for GC–MS analysis. A 2 μL of derivatized sample was injected by an Agilent 4683B GC auto-sampler (Agilent Technologies, Atlanta, USA) into an Agilent 6890N gas chromatograph with 5973 mass spectrometry at 250 °C without split. The detail program was set as previous study (Shi et al., 2015). GC-MS chromatograms were processed using GAVIN based on Matlab (Behrends, Tredwell, & Bundy, 2011). Deconvolution was achieved by AMDIS and the results were imported into GAVIN for retention time correction and area integration across the data set. Identification of metabolites from GC-MS analysis was supported by comparison with a standard mix (Supelco 37 Component FAME Mix; Sigma-Aldrich; six monosaccharide quantitative standards; Ludger).

**Supplementary Figures**

**Figure S1** Rotated principal component analysis of flight muscle. Variance between intra-species represented 59.1%, 13.1%, and 8% for PC1, PC2, and PC3, respectively. Variance between inter-species represented 58.7%, 18.4%, and 6.5% for PC1, PC2, and PC3, respectively. FD, fiber density; FS, fiber area; MD, myofibril diameter; CD, capillary density; CA, capillary area; CF, the number of capillaries per fiber; DS, proportion of subsarcolemmal mitochondrion; Vv .mt., total mitochondrial volume density; Vv .ssm., volume density of subsarcolemmal mitochondrion; Vv .imm., volume density of intermyofibrillar mitochondrion; Vv.sim., the difference between Vv .ssm. and Vv .imm.; Vv .LD.: volume density of lipid droplet.

**Figure S2** 18 and 22 of soft power for scalefree network construction of intraspecies (a) and interspecies (b) respectively. Correlation between co-expression modules and muscle traits across inter- (c) as well as intra-species (d).

**Figure S3** Modules correspond to muscle samples within species (a) and between species (b). Heat maps describing expression levels for all DEGs in muscle for each module, Barplots of the values of the module eigengene (i.e., the ﬁrst principal component) derived from singular value decomposition are displayed for each module.

**Figure S4** Network heatmaps of the 966 and 2457 genes differentially expressed between intraspecies (a) and interspecies (b) respectively. Pearson correlations between expression profiles of all pairs of genes were transformed into network connection strengths. Hub genes are highlighted in black squares.

**Figure S5** Relative levels of intermediates and fatty acids in fasting plasma. * significant increase; ‡ significant decrease.

**Figure S6** Associations between wing length/body length and fiber area (a), between wing length/body length and myofibril diameter (b), between Vv (mt) and Vv (LD) (c), between Vv (mt) and myofibril diameter (d), and between Vv (LD) and myofibril diameter (e).

**Figure S7** Protein levels of *MEF2C* and *EPAS1.*


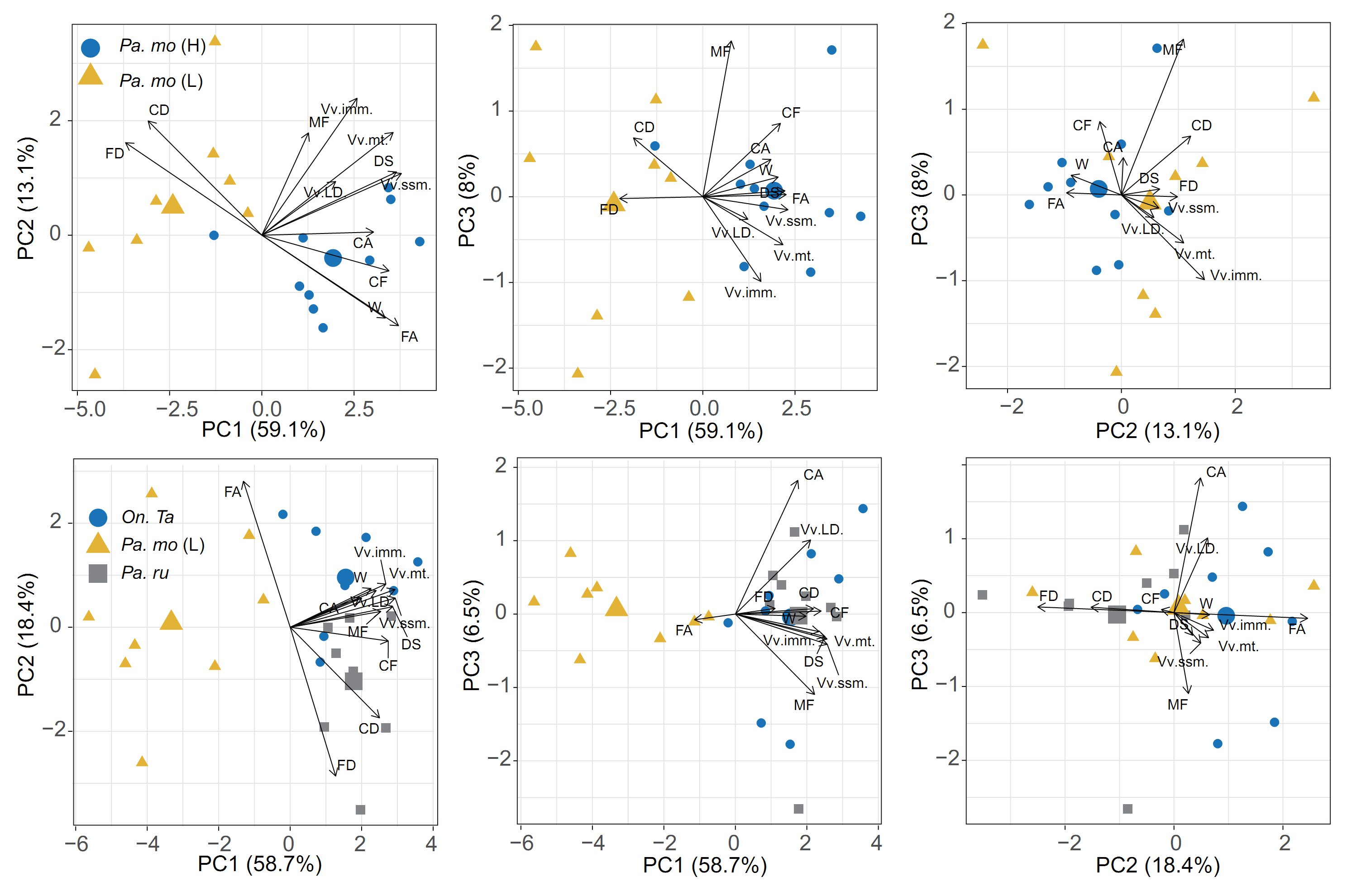


**Figure S1**


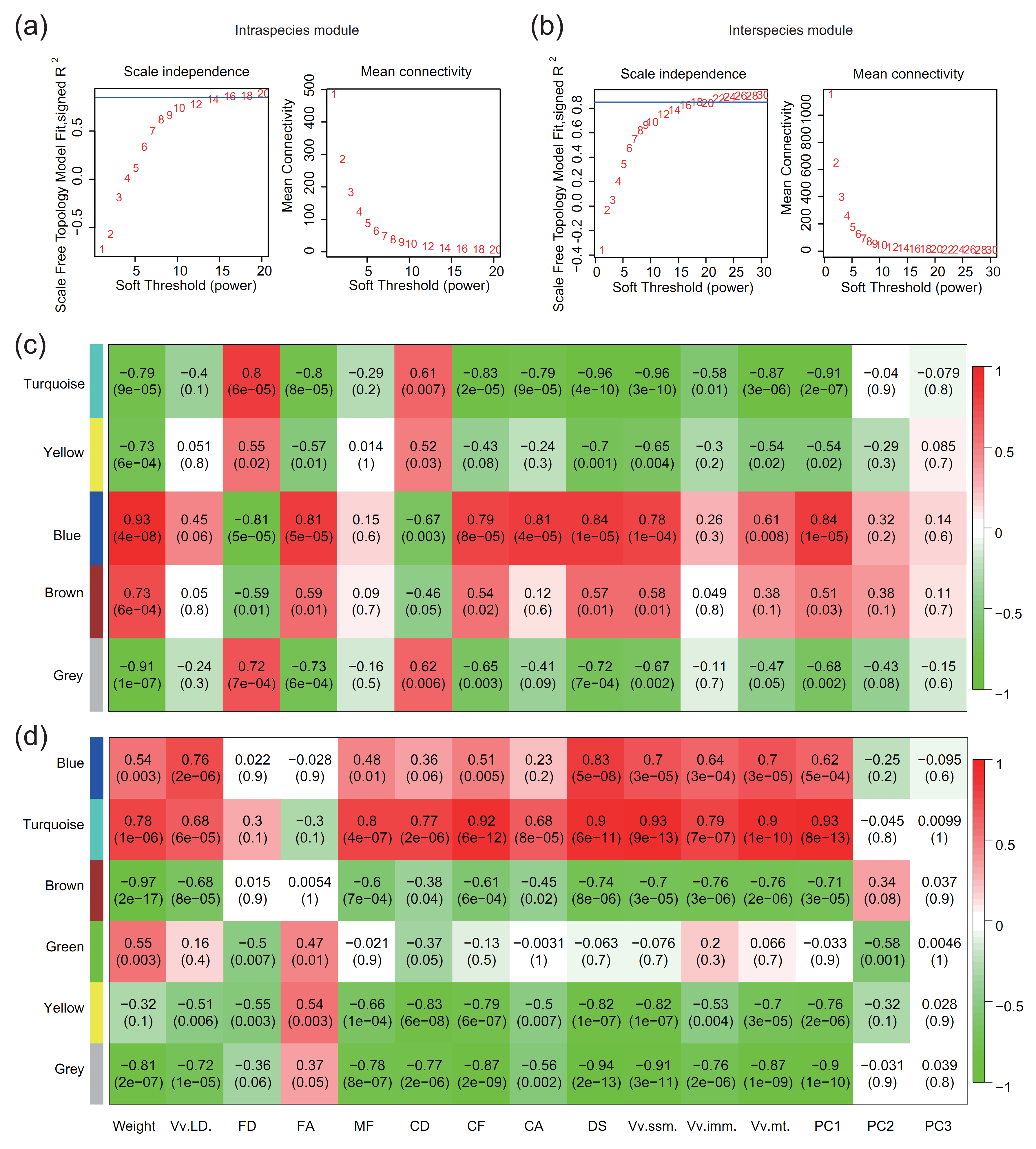


**Figure S2**


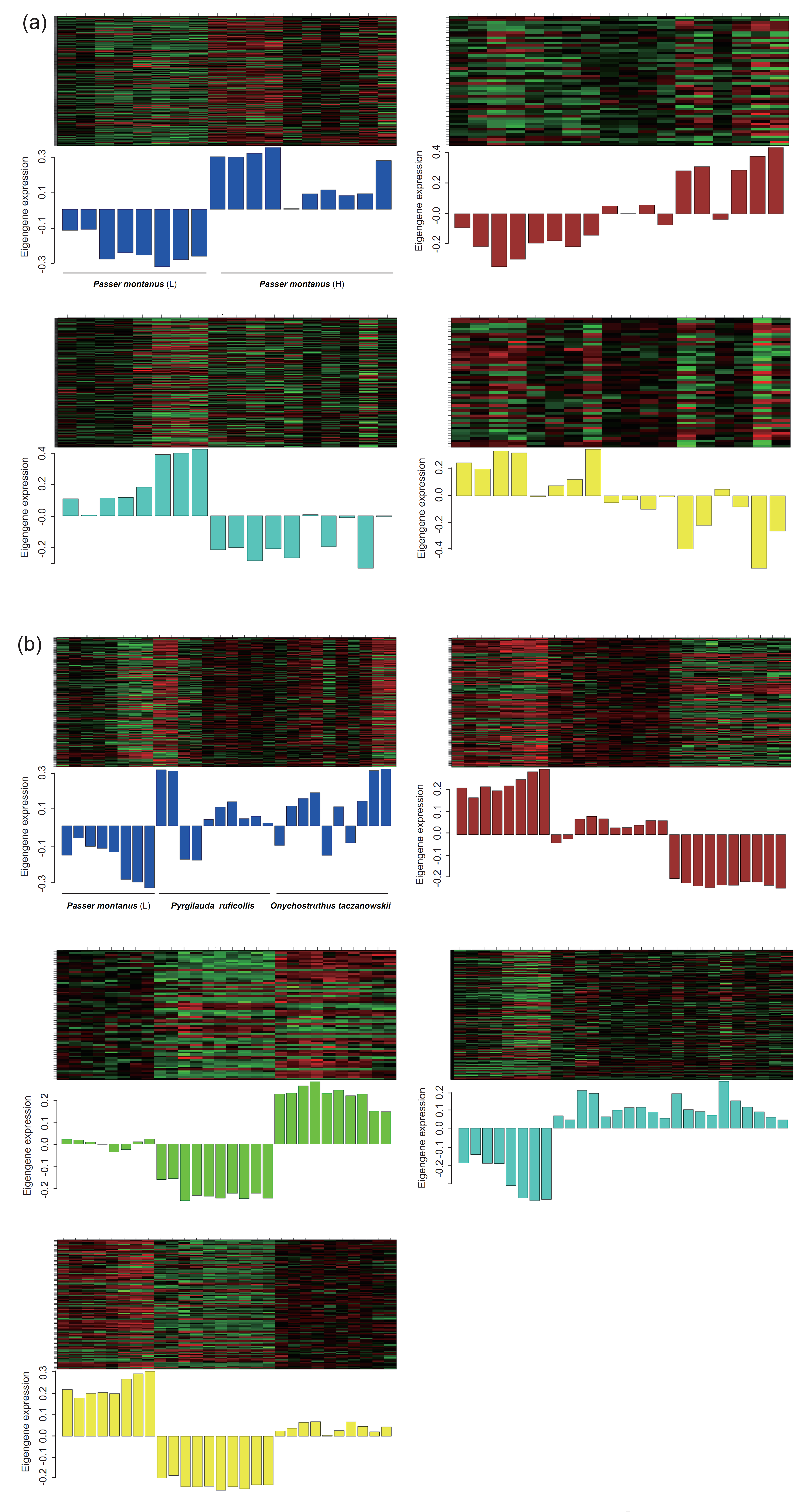


**Figure S3**


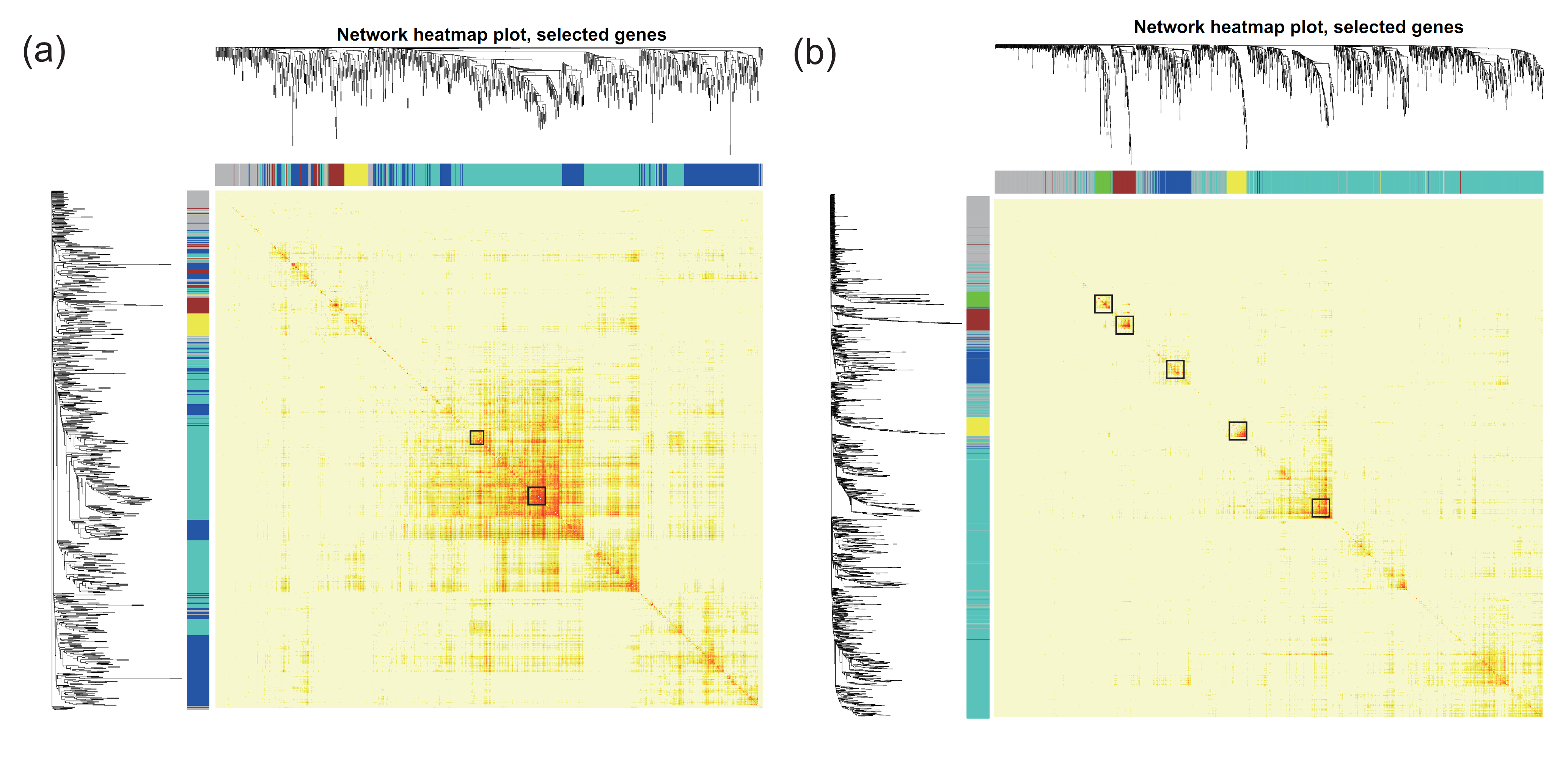


**Figure S4**


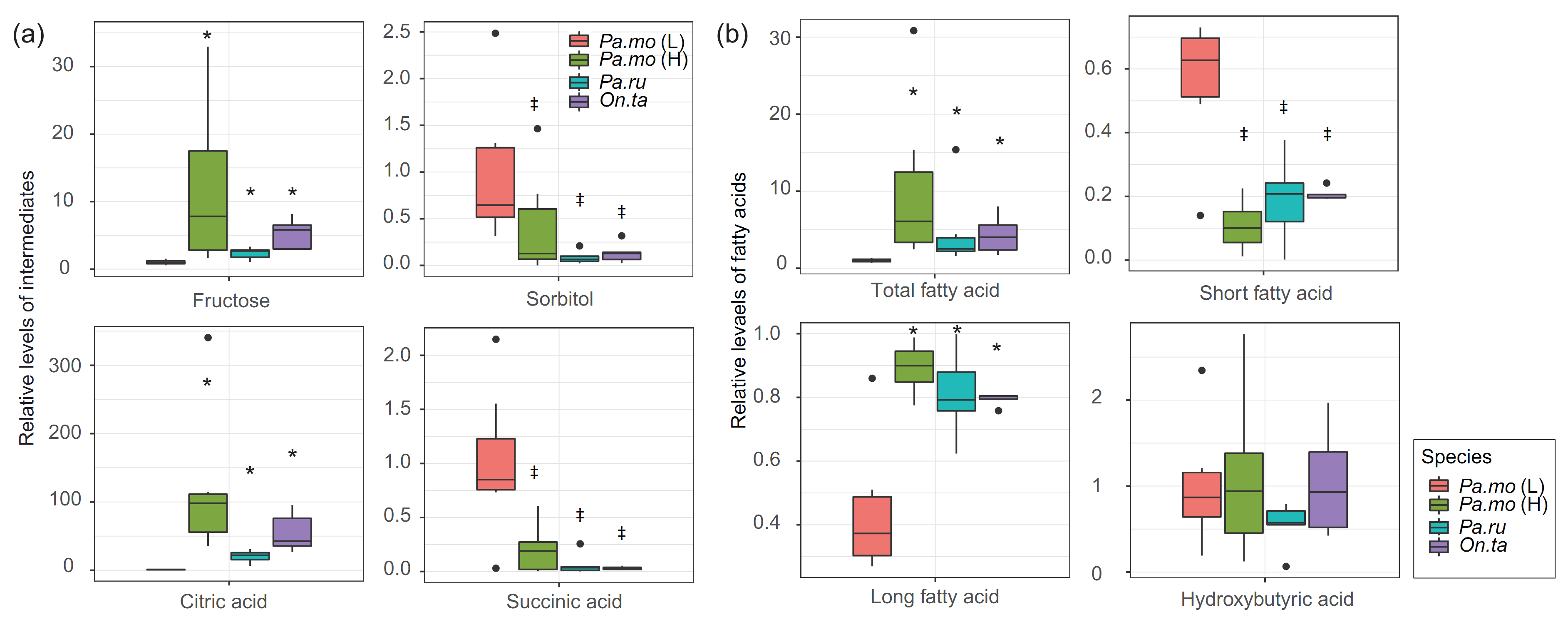


**Figure S5**


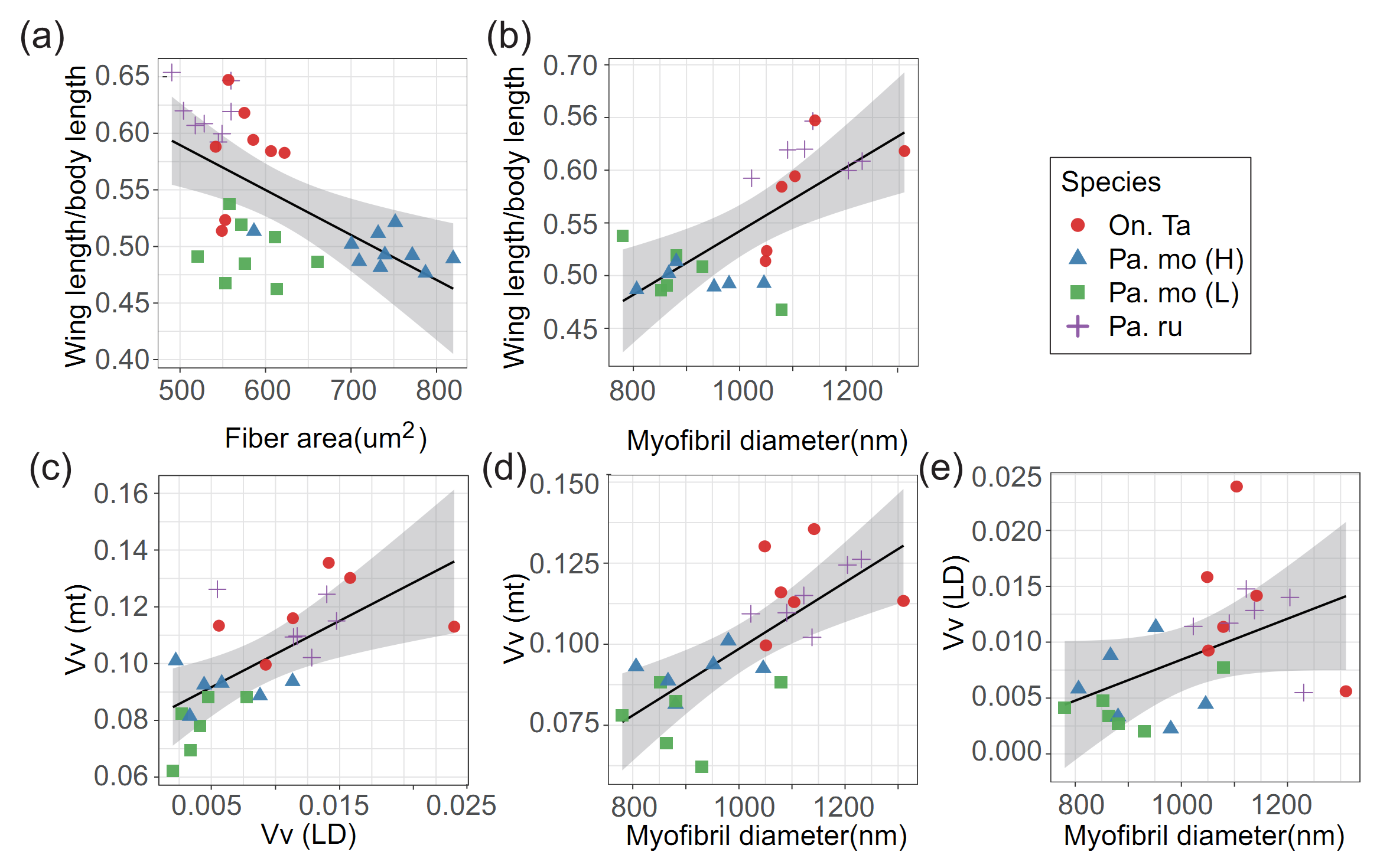


**Figure S6**


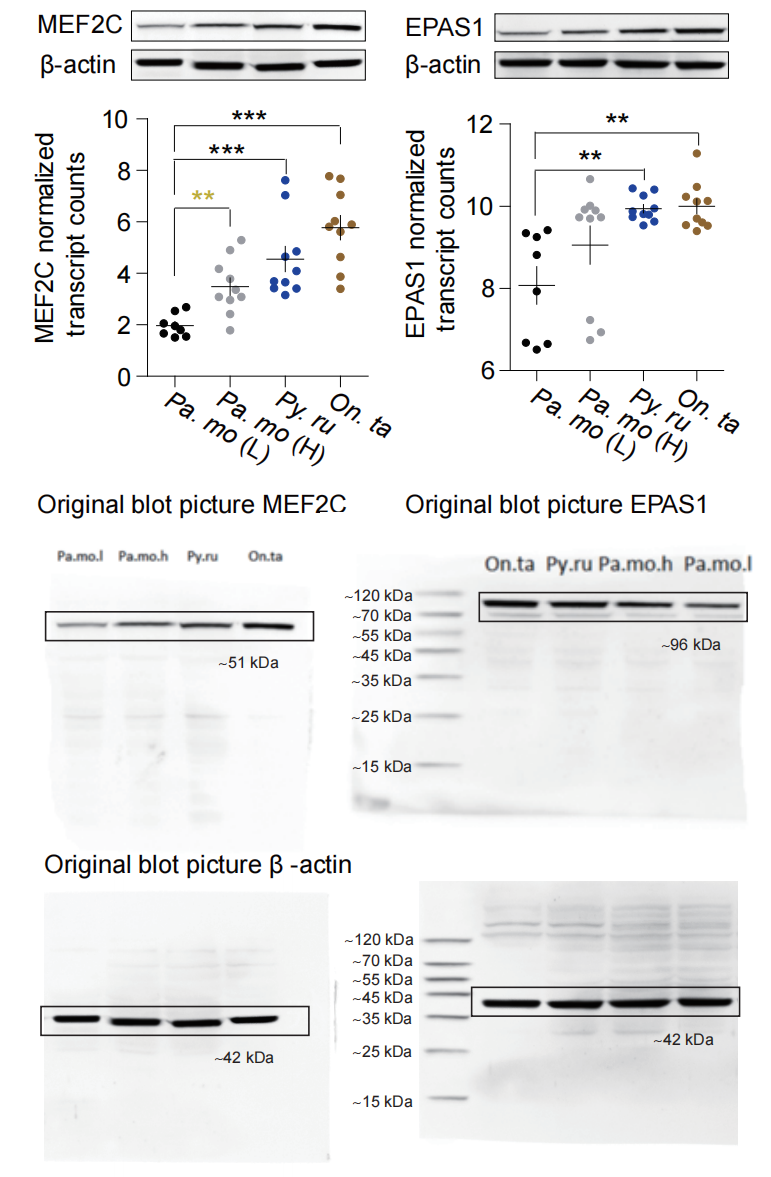


**Figure S7**

**Supplementary tables**

**Table S1** Specimen information used for phenomic, transcriptomic and metabolomic analyses

| **Specimen ID** | **Species** | **Location** | **Sex** | **Histochemistry** | **Electron**  **microscope** | **Transcriptomics** | **Metabolomics** | **Clean Reads** | **Q20** | **Q30** | **GC** | **Overall mapping rate** |
| --- | --- | --- | --- | --- | --- | --- | --- | --- | --- | --- | --- | --- |
| Pamo_L1 | *Pa. mo* | Lowland | M | √ |  | √ | √ | 21874595 | 99.84% | 97.19% | 54% | 92.39% |
| Pamo_L2 | *Pa. mo* | Lowland | F | √ |  | √ | √ | 23585717 | 99.84% | 97.25% | 54% | 91.39% |
| Pamo_L3 | *Pa. mo* | Lowland | M | √ | √ | √ | √ | 26615472 | 99.86% | 97.24% | 54% | 90.87% |
| Pamo_L4 | *Pa. mo* | Lowland | M | √ | √ | √ | √ | 32121798 | 99.85% | 97.37% | 54% | 91.96% |
| Pamo_L5 | *Pa. mo* | Lowland | M | √ | √ | √ | √ | 17166225 | 100.00% | 97.95% | 50% | 92.57% |
| Pamo_L6 | *Pa. mo* | Lowland | M | √ | √ | √ | √ | 20441592 | 100.00% | 97.85% | 51% | 90.19% |
| Pamo_L7 | *Pa. mo* | Lowland | F | √ | √ | √ | √ | 16679631 | 100.00% | 97.61% | 53% | 89.72% |
| Pamo_L8 | *Pa. mo* | Lowland | F | √ | √ | √ | √ | 20923512 | 100.00% | 97.12% | 54% | 87.51% |
| Pamo_L9 | *Pa. mo* | Lowland | F |  |  |  | √ |  |  |  |  |  |
| Pamo_L10 | *Pa. mo* | Lowland | M |  |  |  | √ |  |  |  |  |  |
| Pamo_H1 | *Pa. mo* | Highland | M | √ |  | √ | √ | 30353485 | 99.86% | 97.45% | 54% | 92.58% |
| Pamo_H2 | *Pa. mo* | Highland | M | √ | √ | √ | √ | 30619395 | 99.85% | 97.46% | 54% | 92.59% |
| Pamo_H3 | *Pa. mo* | Highland | M | √ | √ | √ | √ | 28657802 | 99.83% | 97.35% | 54% | 92.45% |
| Pamo_H4 | *Pa. mo* | Highland | F | √ | √ | √ | √ | 26201993 | 99.81% | 97.19% | 55% | 91.06% |
| Pamo_H5 | *Pa. mo* | Highland | F | √ | √ | √ | √ | 16937478 | 100.00% | 97.53% | 53% | 91.68% |
| Pamo_H6 | *Pa. mo* | Highland | M | √ | √ | √ | √ | 18632409 | 100.00% | 97.80% | 51% | 89.62% |
| Pamo_H7 | *Pa. mo* | Highland | F | √ |  | √ | √ | 28465514 | 99.84% | 97.23% | 54% | 91.32% |
| Pamo_H8 | *Pa. mo* | Highland | F | √ | √ | √ | √ | 20693777 | 100.00% | 97.84% | 52% | 89.47% |
| Pamo_H9 | *Pa. mo* | Highland | M |  |  | √ |  | 20686863 | 100.00% | 97.83% | 53% | 90.91% |
| Pamo_H10 | *Pa. mo* | Highland | M |  |  | √ |  | 28392712 | 99.78% | 96.64% | 53% | 88.07% |
| Pamo_H11 | *Pa. mo* | Highland | F | √ |  |  | √ |  |  |  |  |  |
| Pamo_H12 | *Pa. mo* | Highland | M | √ |  |  | √ |  |  |  |  |  |
| Pyru_1 | *Pa. ru* | Highland | F |  |  | √ |  | 23469997 | 99.47% | 93.07% | 49% | 94.58% |
| Pyru_2 | *Pa. ru* | Highland | M |  |  | √ |  | 23375103 | 99.43% | 92.78% | 49% | 93.71% |
| Pyru_3 | *Pa. ru* | Highland | F | √ |  | √ | √ | 30155654 | 99.81% | 96.42% | 53% | 91.04% |
| Pyru_4 | *Pa. ru* | Highland | F | √ | √ | √ | √ | 27808582 | 99.81% | 96.98% | 53% | 93.66% |
| Pyru_5 | *Pa. ru* | Highland | M | √ | √ | √ | √ | 32078129 | 99.86% | 97.35% | 54% | 91.37% |
| Pyru_6 | *Pa. ru* | Highland | F | √ | √ | √ | √ | 32215295 | 99.88% | 97.44% | 53% | 92.20% |
| Pyru_7 | *Pa. ru* | Highland | F | √ |  | √ | √ | 27290142 | 99.85% | 97.31% | 54% | 91.33% |
| Pyru_8 | *Pa. ru* | Highland | M | √ | √ | √ | √ | 26864261 | 99.83% | 97.31% | 53% | 92.53% |
| Pyru_9 | *Pa. ru* | Highland | F | √ | √ | √ | √ | 24000207 | 99.85% | 97.30% | 53% | 92.19% |
| Pyru_10 | *Pa. ru* | Highland | M | √ | √ | √ | √ | 30895151 | 99.87% | 97.49% | 52% | 92.88% |
| Pyru_11 | *Pa. ru* | Highland | M | √ |  |  | √ |  |  |  |  |  |
| Pyru_12 | *Pa. ru* | Highland | F | √ |  |  | √ |  |  |  |  |  |
| Onta_1 | *On. ta* | Highland | F | √ | √ | √ | √ | 25874433 | 99.85% | 96.89% | 54% | 92.33% |
| Onta_2 | *On. ta* | Highland | M | √ | √ | √ | √ | 28284994 | 99.84% | 97.08% | 54% | 91.76% |
| Onta_3 | *On. ta* | Highland | F | √ |  | √ | √ | 28463062 | 99.82% | 97.05% | 55% | 90.42% |
| Onta_4 | *On. ta* | Highland | F | √ | √ | √ | √ | 30084403 | 99.83% | 96.91% | 56% | 89.42% |
| Onta_5 | *On. ta* | Highland | F | √ | √ | √ | √ | 26095232 | 99.81% | 96.94% | 53% | 94.35% |
| Onta_6 | *On. ta* | Highland | F | √ |  | √ | √ | 32056855 | 99.86% | 97.20% | 55% | 92.01% |
| Onta_7 | *On. ta* | Highland | M | √ | √ | √ | √ | 31938798 | 99.88% | 97.32% | 53% | 92.89% |
| Onta_8 | *On. ta* | Highland | F | √ | √ | √ | √ | 30878688 | 99.86% | 97.17% | 53% | 91.45% |
| Onta_9 | *On. ta* | Highland | F |  |  | √ |  | 23592057 | 99.45% | 93.03% | 49% | 93.35% |
| Onta_10 | *On. ta* | Highland | F |  |  | √ |  | 23462484 | 99.47% | 93.10% | 49% | 91.25% |
| Onta_11 | *On. ta* | Highland | F | √ |  |  | √ |  |  |  |  |  |
| Onta_12 | *On. ta* | Highland | M | √ |  |  | √ |  |  |  |  |  |

**Table S3** Gene ontology terms, Human Phenotype Ontology, and KEEG pathways enriched in shared differentially expressed genes

| **GO Term** | **No. of Genes** **(P)** | **KEGG Pathway** | **No. of Genes** **(P)** |
| --- | --- | --- | --- |
| **Shared up-expressed DEGs between snow finches and highland tree sparrow** | | | |
| Muscle structure development | 11(0.0244) | Primary bile acid biosynthesis | 3(0.0079) |
| Lipid metabolic process | 16(0.0404) |  |  |
| Oxygen transport | 3(0.0078) |  |  |
| Myosin complex | 4(0.0172) |  |  |
| **Shared down-expressed DEGs between snow finches and highland tree sparrow** | | | |
| Autophagy | 8(0.0147) | Proteasome | 7(0.0000) |
| Cell death | 23(0.0015) | Lysosome | 5(0.0156) |
| Apoptotic process | 20(0.0064) |  |  |

**Table S4** Gene ontology terms, Human Phenotype Ontology, and KEEG pathways enriched in muscle functional module of differentially expressed genes

| **Module**  **(No. of Genes)** | **PCAs** | **GO Term** | **No. of**  **Genes** **(P)** | **KEGG Pathway** | **No. of Genes (P)** |
| --- | --- | --- | --- | --- | --- |
| **Population network** | | | | | |
|  | | | | | |
| Turquoise(438) | PC1 | Muscle system process | 17(0.006) | Citrate cycle (TCA cycle) | 5(0.037) |
|  |  | Muscle contraction | 15(0.006) | Proteasome | 9(0.000) |
|  |  | Mitochondrion | 30(0.023) |  |  |
|  |  | Carbohydrate derivative metabolic process | 30(0.000) |  |  |
| Blue(300) | PC1 | Muscle structure development | 12(0.000) |  |  |
|  |  | Muscle contraction | 7(0.0281) |  |  |
|  |  |  |  |  |  |
|  |  |  |  |  |  |
| Brown(50) | PC1 | Mitochondrion | 14(0.000) | Oxidative phosphorylation | 5(0.001) |
|  |  | Respiratory chain | 7(0.000) |  |  |
| Yellow(51) | PC1 | Organelle subcompartment | 9(0.030) |  |  |
| **Species network** | | | | | |
|  | | | | | |
| Turquoise(1417) | PC1 | Developmental process | 306(0.000) | Proteasome | 12(0.000) |
|  |  | Muscle structure development | 36(0.000) | Inositol phosphate metabolism | 16(0.001) |
|  |  | Vasculature development | 53(0.000) | Phosphatidylinositol signaling system | 18(0.005) |
|  |  | Mitochondrion | 80(0.000) |  |  |
|  |  | Lipid metabolic process | 66(0.009) |  |  |
|  |  | Abnormality of the vasculature | 53(0.014) |  |  |
| Blue(181) | PC1 | - | - | Insulin signaling pathway | 6(0.043) |
|  |  |  |  |  |  |
|  |  |  |  |  |  |
| Brown(113) | PC1 | Organelle | 72(0.009) |  |  |
|  |  | Intracellular organelle | 71(0.009) |  |  |
| Yellow(103) | PC1 | Cellular_component | 71(0.033) |  |  |
|  |  | Membrane-bounded organelle | 56(0.000) |  |  |
| Green(68) | PC2 | Membrane part | 15(0.040) |  |  |

**Table S6** Candidate DEGs connected with phenotypic variations

| **Phenotype** | **Positive association** | **Negative association** |
| --- | --- | --- |
| Fiber density | *HDAC3*, MEF2C*, SMAD6* | *MYBPH* MYOZ3, MSTN* |
| Myofibril density | *SAMD6*, HDAC3*, EPAS1** | *MRF4* |
| Capillary density | *VEGFR2*, VEGFR3*, TIE2*, DLL4*, NOTCH2, FGFR4*, ANGPT1, HEY2** |  |
| Capillary to fiber ratio | *EPAS1*, VEGFR4*, VEGFR2*, VEGFR3*, TIE2*, DLL4*, NOTCH2*, FGFR4*, ANGPT1, HEY2** | *VEGFD** |
| Capillary area | *EPAS1*, VEGFR4*, VEGFR2*, VEGFR3*, TIE2, DLL4*, NOTCH2*, FGFR4, ANGPT1, HEY2, LAMA4*, FLVC1, SEMA3C* |  |
| Proportion of subsarcolemmal mitochondrion | *EPAS1*, TRAK2*, PGC1α*, CLUH* |  |
| Total mitochondrial volume density | *EPAS1*, PGC1α, MIGA2*, MIGA1, MID51** | *BNIP3*, WIPI2*, LC3, FUNDC1*, MUL1** |
| Volume density of lipid droplet | *DGAT2*, EPAS1*, LIPC*, PLA2G3*, CPT1a** | *PPARδ* |

*Association that remained signiﬁcant after Bonferroni correction for multiple comparisons
